# Structure-Based Optimization of 1,2,4-Triazole-3-Thione Derivatives: Improving Inhibition of NDM-/VIM-Type Metallo-β-Lactamases and Synergistic Activity on Resistant Bacteria

**DOI:** 10.3390/ph16121682

**Published:** 2023-12-02

**Authors:** Matteo Bersani, Mariacristina Failla, Filippo Vascon, Eleonora Gianquinto, Laura Bertarini, Massimo Baroni, Gabriele Cruciani, Federica Verdirosa, Filomena Sannio, Jean-Denis Docquier, Laura Cendron, Francesca Spyrakis, Loretta Lazzarato, Donatella Tondi

**Affiliations:** 1 Department of Drug Science and Technology, University of Turin, Via Pietro Giuria 9, 10125 Turin, Italy; matteo.bersani@unito.it (M.B.); mariacristina.failla@unito.it (M.F.); eleonora.gianquinto@unito.it (E.G.); francesca.spyrakis@unito.it (F.S.); 2 Department of Biology, University of Padua, Viale G. Colombo 3, 35121 Padua, Italy; filippo.vascon@unipd.it (F.V.); laura.cendron@unipd.it (L.C.); 3 Department of Life Sciences, University of Modena and Reggio Emilia, Via Campi 103, 41125 Modena, Italy; laura.bertarini@unimore.it; 4 Kinetic Business Centre, Molecular Discovery Ltd., Elstree, Borehamwood, Hertfordshire WD6 4PJ, UK; massimo@moldiscovery.com; 5 Department of Chemistry, Biology and Biotechnology, Università Degli Studi di Perugia, Via Elce di Sotto, 06132 Perugia, Italy; gabriele.cruciani@unipg.it; 6 Department of Medical Biotechnologies, University of Siena, Viale Bracci 16, 53100 Siena, Italy; federica.verdirosa95@gmail.com (F.V.); filomena.sannio@unisi.it (F.S.); jddocquier@unisi.it (J.-D.D.); 7 Laboratoire de Bactériologie Moléculaire, Centre d’Ingénierie des Protéines-InBioS, Université de Liège, B-4000 Liège, Belgium

**Keywords:** 1,2,4-triazole-3-thione, structure-based drug design, NDM-1, competitive inhibitors, resistance

## Abstract

The worldwide emergence and dissemination of Gram-negative bacteria expressing metallo-β-lactamases (MBLs) menace the efficacy of all β-lactam antibiotics, including carbapenems, a last-line treatment usually restricted to severe pneumonia and urinary tract infections. Nonetheless, no MBL inhibitor is yet available in therapy. We previously identified a series of 1,2,4-triazole-3-thione derivatives acting as micromolar inhibitors of MBLs in vitro, but devoid of synergistic activity in microbiological assays. Here, via a multidisciplinary approach, including molecular modelling, synthesis, enzymology, microbiology, and X-ray crystallography, we optimized this series of compounds and identified low micromolar inhibitors active against clinically relevant MBLs (NDM-1- and VIM-type). The best inhibitors increased, to a certain extent, the susceptibility of NDM-1- and VIM-4-producing clinical isolates to meropenem. X-ray structures of three selected inhibitors in complex with NDM-1 elucidated molecular recognition at the base of potency improvement, confirmed in silico predicted orientation, and will guide further development steps.

## 1. Introduction

The worldwide rise of antibiotic resistance, especially in Gram-negative bacteria, is a threatening public health problem that needs to be addressed to mitigate mortality, morbidity, and socio-economic burden [1,2].

WHO has identified “critical priority” organisms against which the identification of new antibacterial drugs is extremely desirable. Among these stand the superbug multidrug-resistant *Klebsiella pneumoniae*, the carbapenem-resistant *Acinetobacter baumannii,* and the multidrug-resistant *Pseudomonas aeruginosa* [3,4,5,6]. 

Resistance to β-lactams, the most widely used class of antibiotics in antibacterial chemotherapy, thus represents a serious challenge [7,8]. Resistance to β-lactams is mediated, especially in Gram-negative spp., by the production of β-lactamase enzymes (BLs) that catalyze hydrolysis of the β-lactam ring. Based on their structural and mechanistic properties, BLs are classified in four molecular classes (A, B, C, and D) and three functional groups. Class A, C, and D include active serine β-lactamases (SBLs), while class B corresponds to metallo-β-lactamases (MBLs). Carbapenems, an important subfamily of β-lactams representing an important therapeutic solution for the treatment of infections caused by MDR isolates, are inactivated by the so-called carbapenemases, whose epidemiological relevance is increasing worldwide. They include the class A KPC- and the class D OXA-48-type serine carbapenemases, and MBLs. 

MBL enzyme subtypes (e.g., IMP, VIM, NDM) encoded by genetic mobile elements have emerged and disseminated rapidly worldwide, in organisms of high clinical relevance, such as *Enterobacterales* and *P. aeruginosa* [9,10,11]. The worldwide spread of bacterial strains producing clinically relevant MBLs, not susceptible even to last resort carbapenems, largely involves Europe as well [12], and patients colonized with such isolates are common in Italy [13].

Structurally, MBLs are classified in three subclasses (B1–B3) and all share the αβ/βα fold typical of the metallo-hydrolase/oxydoreductase superfamily [9]. However, MBLs are highly divergent and the differences among the active site architectures, the nature of zinc ligands, and the catalytic mechanisms have limited the development of broad-spectrum inhibitors, or even compounds targeting the same subclass. 

Subclass B1 has been the focus of our work, as it includes the most widespread and clinically relevant MBLs, among which is NDM-1. All members of the B1 subclass possess a shallow active-site groove containing two catalytic divalent zinc ions, flanked by the flexible loops L3 and L10. In contrast to other MBLs in the B1 subgroup, NDM-1 is lipidated and anchored to the inner leaflet of the outer membrane of Gram-negatives, a unique subcellular localization that was shown to increase its stability to zinc deprivation and that promotes its secretion in outer membrane vesicles [14,15,16]. Plasmids carrying NDM-1 genes are often associated with additional resistance markers encoding other antibiotic resistance mechanisms, whose acquisition determines an evolution towards ultra-resistant phenotypes [17]. As a consequence, clinical strains producing NDM-1 are only susceptible to last-line antibacterial agents, like colistin, tigecycline, or fosfomycin, all of which have toxicity limitations [18,19,20]. 

Several NDM-1 inhibitors have been reported over the years, with only a few reaching clinical trials, nevertheless with swinging results [21]. The development of a potent NDM-1 inhibitor, ideally able to target other relevant B1 MBLs, represents a real demand and will be crucial for the effective fight against bacterial resistance: such an inhibitor would preserve the efficacy of β-lactams and would restore the susceptibility of Gram-negative bacteria toward previously used β-lactams.

Among the several classes of molecules under study as potential MBL inhibitors, triazole-thione compounds are a well-known class of molecules [22,23,24,25,26]. We recently explored the potential of such chemistry and developed 1,2,4-triazole-3-thione-based MBL inhibitors [27]. At that time, the analysis of interactions of the developed compounds with MBLs evidenced the critical role of the pharmacophore triazole thiolate in interacting with zinc ions in the active site. The predicted binding orientation in MBLs showed the triazole nitrogen in position 2 and the thiolate coordinating Zn1 and Zn2, respectively, thus acting as the metal binding pharmacophore. Other than the Zn coordination, the interactions within the catalytic pocket were mainly hydrophobic and involved residues located in the L3 loop, overall stabilizing the ligand in the binding site. 

Here, we aim to improve both the inhibitory potency and the spectrum of activity of rationally designed MBL inhibitors based on a computational chemistry approach and thorough analysis of the NDM-1 active site. In silico design, carried out using the enzyme NDM-1 in the first instance, guided the selection of possible modulations at the benzylidene ring and at the 5-position of the triazole ring. In the newly obtained library, various substituents were selected and introduced on the benzylidene ring to modulate steric hindrance and electronic properties. Different hydrophobic moieties were selected and introduced in the 5-position, to increase interactions with the flexible loops (L3/L10) that are proximal to the catalytic site and involved in substrate recognition. 

The implementation of the above-mentioned approach allowed the identification of promising compounds, whose interaction with relevant MBLs was thoroughly investigated by X-ray crystallography, biochemical methods, and microbiological assays.

## 2. Results and Discussion

### 2.1. Molecular Interaction Field (MIF) Analysis

**Figure 1:**
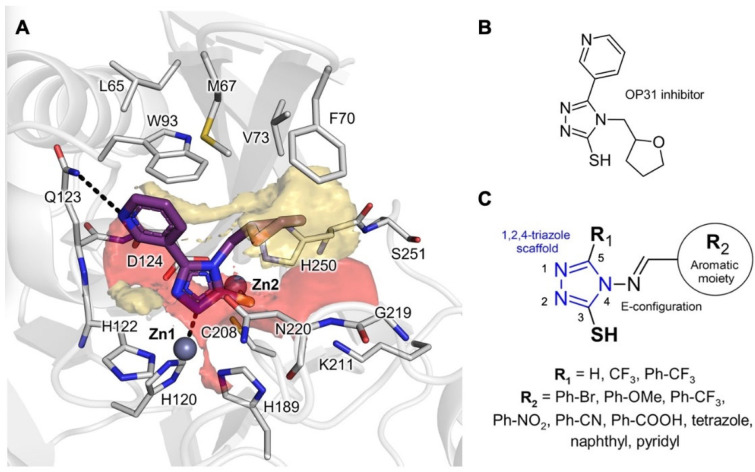
Molecular Interaction Field (MIF) analysis. (**A**) MIF contours of NDM-1 binding site (PDB ID: 6TGD). Red contour (O MIF) and yellow contour (DRY MIF), respectively, show binding site regions in which H-bond acceptors/electron-rich group and hydrophobic moieties can favorably interact with NDM-1 residues; (**B**) molecular structure of previous OP31 inhibitor co-crystallized in NDM-1 (PDB ID: 6TGD); (**C**) modulations introduced on 1,2,4-triazole scaffold for generating the reported library. The protein is shown in cartoons; the ligand and the residues lining the pocket are displayed in capped sticks. Hydrogen and coordination bonds are shown as dashed black lines.

The ligand binding site of NDM-1 consists of a pocket located at the interface between two five-stranded beta-sheets, enclosing the two catalytic zinc ions and delimited by the flexible L3 and L10 loops. The analysis of the corresponding Molecular Interaction Fields (MIFs) for NDM-1 structure (6TGD), previously published by our group [24], confirmed the hydrogen bond acceptor character of the pocket, mainly associated with the presence of the zinc ions, the key positively charged Lys211 residue on the L10 loop, and the several NH backbone groups facing the catalytic site (Figure 1A). A highly hydrophobic region made up by hydrophobic residues of the. L3 loop (Leu65, Met67, Phe70, Val73, Trp93) can also be identified. The flexibility of Gln123 and Asn220, observed when analyzing different X-ray structures, might slightly modify MIFs’ extension among different MBLs, without changing the general electronic properties of the binding site.

We chose to keep the 1,2,4-triazole-3-thione/thiol core and the benzylidene moiety from our previous study (Figure 1B) [27], to strike a balance between the necessary hydrophobicity and the electron-rich nature of NDM-1 inhibitors. We, thus, explored a variety of substituents to the aromatic benzylidene ring and the 5-position of the triazole ring (Figure 1C). First, hydrophobic and electron-rich substituents as bromine, methoxyl, trifluoromethyl, nitro, cyano, carboxyl, or tetrazole groups were added to vary steric hindrance and electronic properties of the benzylidene moiety. Then, hydrophobic trifluoromethyl and (4-trifluoromethyl)-phenyl moieties were inserted at the 5-position of the triazole core to contact the hydrophobic L3 loop, similarly to what was observed for the N-acyl substituents in penicillin and cephalosporin substrates [28]. 

### 2.2. Chemistry

**Scheme 1:**
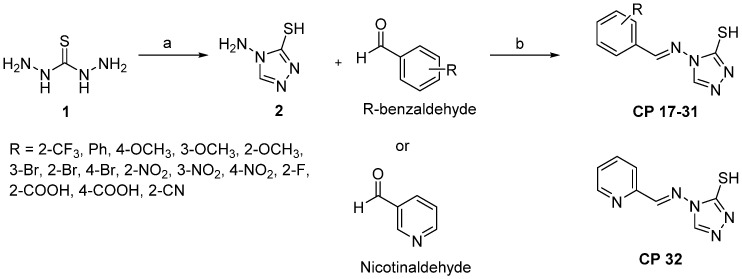
Synthesis of intermediate **2** and compounds **CP 17-32**. (a) HCOOH, H_2_O, reflux, 5 h, 43%. (b) CH_3_COOH, reflux, 2–4 h, 27–89%.

The 4-amino-4H-1,2,4-triazole-3-thiol (**2**) intermediate was synthesized according to study [29]. Briefly, it was obtained with a reaction of commercially available thiocarbohydrazide **1** in aqueous formic acid at reflux. A following recrystallization from EtOH yielded a highly pure product, which reacted with the required aromatic aldehydes in excess of acetic acid to obtain target compounds **CP 17–32** (Scheme 1, Table S1). 

**Scheme 2:**
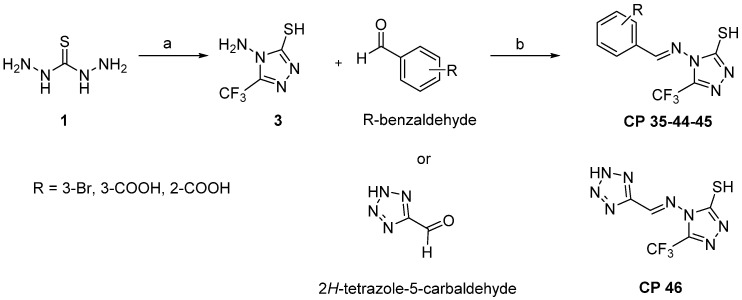
Synthesis of intermediate **3** and compounds **CP 35**, **CP 44-46**. (a) CF_3_COOH, H_2_O, reflux, 5 h, 63%. (b) CH_3_COOH, reflux, 2–4 h, 48–56%.

The 4-amino-5-(trifluoromethyl)-4H-1,2,4-triazole-3-thiol (**3**) intermediate was obtained with a reaction of commercially available thiocarbohydrazide **1** in aqueous trifluoroacetic acid at reflux [30]. A following recrystallization from H_2_O yielded a highly pure product, which reacted with the required aromatic aldehydes in excess of acetic acid to obtain target compounds **CP 35, CP 44-45-46** (Scheme 2, Table S1). 

**Scheme 3:**
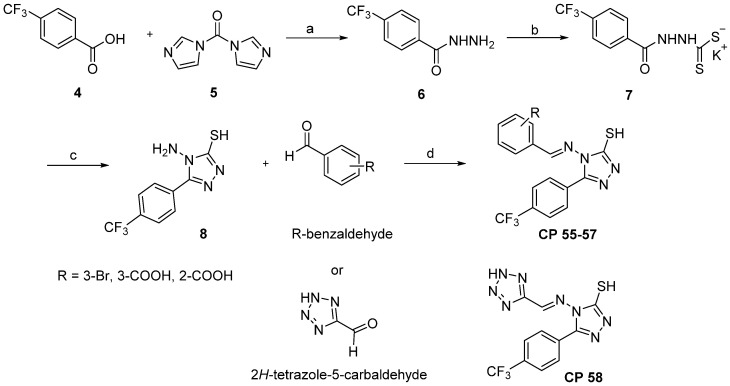
Synthesis of intermediate **8** and compounds **CP 55**–**58**. (a) Dry THF, r.t., 3 h; NH_2_NH_2_*H_2_O, dry THF, r.t., 18 h, 84%. (b) KOH, CS_2_, EtOH, r.t., overnight. (c) NH_2_NH_2_*H_2_O, H_2_O, reflux, 6 h, 84%. (d) CH_3_COOH, reflux, 2–5 h, 12–43%.

The 4-amino-5-(4-(trifluoromethyl)phenyl)-4H-1,2,4-triazole-3-thiol (**8**) intermediate was obtained following the steps shown in Scheme 3 according to study [30]. Briefly, 4-trifluoromethylbenzoic acid was activated using a reaction with 1,1′-carbonyldiimidazole in dry THF. The following reaction with hydrazine monohydrate gave hydrazine derivative **6**. Then, **6** was reacted with CS_2_ in the presence of KOH, and in EtOH as a solvent to give 2-(4-(trifluoromethyl)benzoyl)hydrazine-1-carbodithioate derivative **7** as potassium salt. **7** was then refluxed with hydrazine monohydrate in H_2_O. The desired derivative **8** was isolated with filtration following acidification with HCl. Condensation of **8** with the required aldehydes, in acetic acid at reflux gave final compounds **CP 55**-**58** (Scheme 3, Table S1).

All derivatives were purified and obtained at >95% purity. They were fully characterized by ^1^H-NMR (600 MHz), ^13^C-NMR (150 MHz), and ESI-MS (see Supplementary Materials ).

### 2.3. Molecular Modelling 

**Figure 2:**
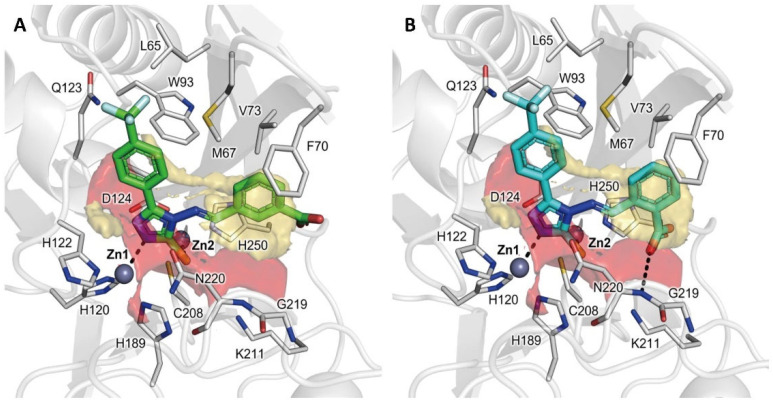
Docking poses of **CP 56** and **CP 57** (**A**,**B**), two representative compounds of the designed library in NDM-1 in PDB ID 6TGD, upon removal of the cognate ligand. The protein is shown in cartoons; the ligand and the residues lining the pocket are displayed in capped sticks. Hydrogen and coordination bonds are shown as dashed black lines.

The synthesized compounds were docked in NDM-1 with the newly developed FlapGlue (Molecular Discovery Ltd., Borehamwood, UK) to validate the MIF-based indications and predict the corresponding binding modes. Most of the compounds, as expected, showed a binding mode similar to that already observed for other 1,2,4-triazole-3-thione/thiol-based compounds [23,24,25,31] (for docking validation procedure, see *Materials and Methods*, Section 3.1). Interestingly, the best poses, in terms of stability of the ligand orientation, number of established interactions, and docking score, were provided by compounds **CP 56** and **CP 57** (Figure 2A,B). Both molecules can coordinate Zn1 and Zn2 ions with the nitrogen of the 1,2,4-triazole ring at the 2-position and the deprotonated thiol group, respectively. Moreover, the carboxyl group of **CP 57** is H-bonded to the Asn220 backbone and interacts through a salt bridge with Lys211 in the identified polar region of the pocket (Figure 1A), while lipophilic imino benzylidene and (4-trifluoromethyl)-phenyl moieties interact with L3 loop residues in the hydrophobic part of the binding site. In contrast, other more hydrophobic and smaller inhibitors only maintained the coordination between the negatively charged sulfur and Zn2.

### 2.4. In Vitro Enzyme Inhibition Assays

The synthesized compounds were all tested for their inhibitory activity against targeted MBLs using a spectrophotometric assay (Table S2). After an initial validation at fixed concentration, compounds yielding > 0% inhibition were profiled in dose–response experiments to determine their *IC*_50_. The *IC*_50_ values were determined by measuring the rate of hydrolysis of the reporter substrate meropenem MEM ([S] = 57 μM; *K*_m_ = 63.4 μM) in the presence of five different inhibitor concentrations. Assays were run after a 5 min incubation. The *K_i_* was either calculated by the Cheng–Prusoff equation assuming competitive inhibition [32], or, for selected inhibitors, fully determined with Dixon plots (Figure S1). Compounds’ evaluation was extended to a panel of other representatives of the subclass B1 MBL enzymes: VIM-1 (substrate: nitrocefin; [S] = 23 μM; *K_m_* = 22 μM), VIM-2 ([S] = 17 μM; *K_m_*= 8.8 μM), and IMP-1 ([S] = 57 μM; *K_m_* = 64.4 μM) (Table 1 and 2, Table S2).

Throughout the series of compounds derivatized on the benzylidene ring (**CP 17**–**32,** Table 1, Table S1), the most active analogues against NDM-1 were the naphthalene derivative **CP 18** and the bromine derivatives **CP 22**, **CP 23**, and **CP 24**, as reported in Table 1. Not all introduced substituents produced activity improvement: for the two carboxylic derivatives **CP 29** and **CP 30**, surprisingly, no inhibitory activity was detected at 100 μM, while for **CP**–**17**, the determined *IC*_50_ was >100 μM. For derivative **CP 22**, we confirmed a competitive inhibition mechanism and determined a *K_i_* of 42.7 μM. Overall, the data obtained for this first series (**CP 17**–**32**, Table S2) allowed the validation of the 1,2,4-triazole-3-thiol analogs as inhibitors of NDM-1; the range of affinity detected vs. NDM-1 closely resembled that already reported vs. VIM-1 for previous similar derivatives [27]. The lack of increase in affinity clearly showed how the single benzylidene ring, even if substituted, is not sufficient to fully complement the NDM-1 binding site. We, thus, explored other potential substitutions to specifically address the hydrophobic region specified by L3.

Indeed, compounds **CP 35**, **CP 44**–**46**, and **CP 55**–**58** carry a hydrophobic moiety at the 5-position, particularly **CP 35** and **CP 44**–**46** present a trifluoromethyl group in the 5-position, while **CP 55**–**58** include a bulkier (4-trifluoromethyl)-phenyl group in their structure. The introduced chemical modifications improved compounds’ activity against NDM-1, and in most cases, maintained or improved the affinity against VIM-2 as well. In fact, most of them maintained a micromolar inhibitory potency against NDM-1 and VIM-2 (*K*_i_ from 4.1 μM to 220 μM) with the only exception of compound **CP 45**, which resulted in inactivity vs. VIM-2. Among the series, compounds **CP 35**, **CP 56**, and **CP 57** acted as potent micromolar inhibitors against both targeted MBLs, likely confirming their capability of filling MBLs’ B1 binding site, as suggested by molecular docking simulations (Table 2). 

The analysis of the results obtained for compounds derivatized at the triazole 5-position (**CP 35**, **CP 44**-**46**, and **CP 55**-**58**) with respect to their non-substituted homologues led to interesting observations on the substituent role. In the case of **CP 35**, for instance, the introduction of a trifluoromethyl moiety returned a compound with improved activity (*K*_i_ = 42.7 μM for **CP 22** vs. *K_i_* = 25.8 μM for **CP 35**). The same happened for **CP 45** (*K_i_* = 83 μM) with respect to **CP 29**, showing no activity. However, the introduction of a bulkier p-trifluoromethylphenyl substituent, as in **CP 55**, returned an eight-fold decrease in activity with respect to **CP 35** (*K*_i_ =25.8 μM for **CP 35** vs. *K*_i_ = 220 μM for **CP 55**). **CP 44** and **CP 46** maintained a similar potency towards NDM-1. Interestingly, **CP 56** and **CP 57**, homologues of **CP 44** and **CP 45**, but bearing the 4-trifluoromethylphenyl group, instead of the trifluoromethyl in the 5-position, demonstrated to be among the best inhibitors of the series. In particular, **CP 57**, the ortho-carboxylic analog, stands out showing the highest potencies against NDM-1 and VIM-2 (*K_i_* values of 5.6 and 4.1 μM, respectively, four- and three-fold more active than **CP 56**), suggesting a more effective and specific interaction for **CP 57** compared to compound **CP 56**, as also suggested by docking simulations (Figure 2). Finally, **CP 58** lost potency and provided a similar activity to the less bulky **CP 46** homologue, possibly because of the absence of a carboxylic group.

Considering the higher structural similarity between NDM-1- and VIM-type BLs than between other subclass B1 MBLs (e.g., IMP-1), the most active compounds were also tested towards VIM-2. In general, the results obtained with VIM-2 agreed with those of NDM-1. Indeed, the most active compounds were not only **CP 35**, **CP 56**, and **CP 57**, but also included **CP 44** and **CP 58**, being active in the micromolar range (Table 2). 

Compounds were also tested on IMP-1 but weak or no inhibitory activity was detected at 200 μM, suggesting different structural requirements to observe significant IMP-1 inhibition, or at least not compatible with the substituents introduced in our chemical series, leading to improved inhibition against NDM-1 and VIM-2, as previously mentioned. 

### 2.5. X-ray Crystallography

**Figure 3:**
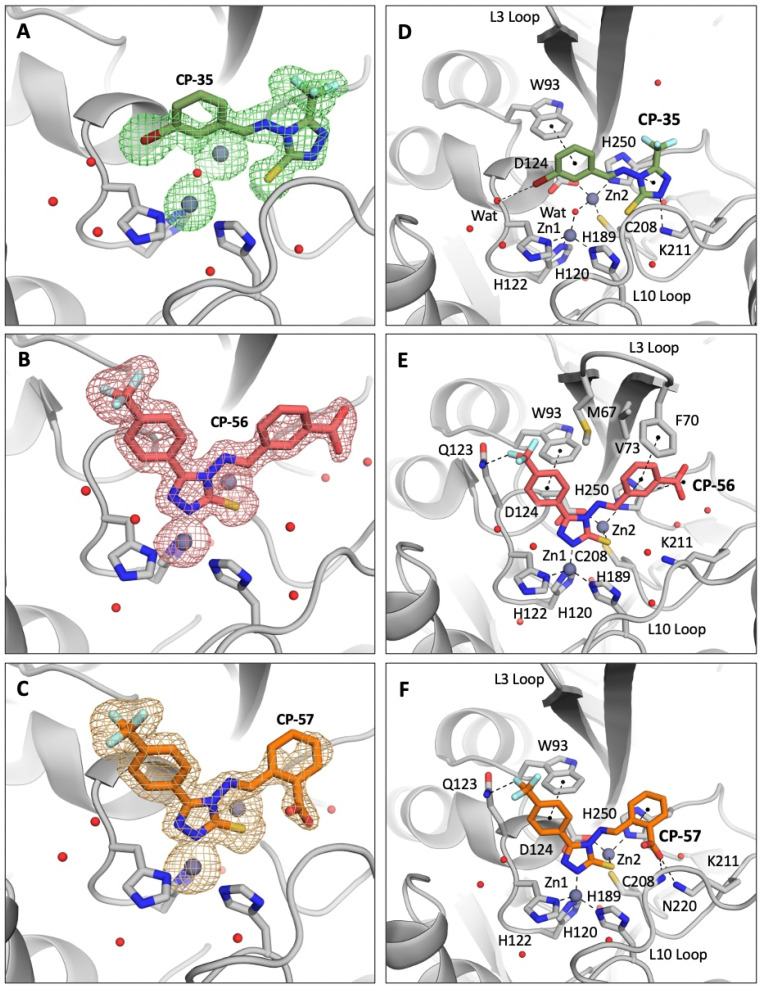
Crystallographic analyses reveal the binding mode of **CP 35, CP 56**, and **CP 57** with NDM-1 (PDB 8B1W, 8B1Z, and 8B20). Omit maps at 2.5 Å confirm the presence of the compounds in NDM-1 active site (**A**–**C**). Binding determinants of the three inhibitors to NDM-1 active site are shown in panels (**D**–**F**).

High-resolution structures of NDM-1 in complex with the most active inhibitors (**CP 35**, **CP 56**, and **CP 57**) were obtained by X-ray crystallography (Table S3). In all the structures, two NDM-1 chains are visible in the asymmetric unit from residue Glu40 to Arg270. As confirmed by omit maps at 2.5 σ (Figure 3A,B), **CP 35** and **CP 56** have been noticeably detected in the active site of both NDM-1 chains (crystallographic occupancy 0.8 and 0.7–0.8, respectively, and RSCC ≥ 0.9). Conversely, **CP 57** is clearly resolved only in the active site of chain A (with crystallographic occupancy of 0.8 and RSCC of 0.92, Figure 3C), while only traces of its presence are detectable in the electron density maps of the chain B catalytic pocket. 

As previously suggested by molecular docking simulations (Figure 2), the phenyl-1,2,4-triazole-3-thiol core of **CP 56** and **CP 57** assumes the same position in the NDM-1 active site of previously developed analogous competitive inhibitors, characterized by the coordination of the two zinc ions by the thiolate group and one of the triazole nitrogens. Furthermore, the 5-(4-(trifluoromethyl)phenyl) group of these inhibitor compounds is stabilized by π-stacking interactions with the Trp93 sidechain and by a hydrogen bond between one of the fluoride atoms and Gln123 Nε2 (Figure 3E,F).

Other π-stacking interactions are established between the His250 imidazole ring and the 3-carboxyphenyl and 2-carboxyphenyl moieties of **CP 56** and **CP 57**, respectively. Despite conserving a high intrinsic flexibility, the L3 loop of NDM-1 in complex with **CP 56** assumes a slightly “closer” conformation than in the complex with **CP 57**, with residues Met67, Phe70, and Val73 defining a hydrophobic pocket that hosts the **CP 56** meta-carboxyphenyl moiety (Figure 3E,F). **CP 57** carboxylate in the ortho-position establishes a salt bridge with the Lys211 side chain (3.5 Å) and a hydrogen bond with Asn220 backbone nitrogen (3.0 Å), while the meta substituent in **CP 56** is not able to reach these residues close enough to produce stabilizing interactions (4.9 Å from Lys211 N_ζ_; 9.1 Å from Asn220 N). These considerations on the position of the carboxylate can explain the higher affinity of **CP 57** for NDM-1 compared to its structural isomer **CP 56**. Noteworthily, NDM-1 Lys211, a residue highly conserved in all subclass B1 representatives apart from the VIM variants, is involved in β-lactam recognition and hydrolysis [21,33,34], and MBL inhibitors that target this residue by carboxylic groups have already displayed a notable activity [26]. In VIM variants, the key contribution in β-lactam binding delivered by Lys211 is picked up by Arg228 [35], suggesting that the ability of the identified best candidates to target these residues might extend their inhibition profile towards different MBLs belonging to the B1 subclass [36]. 

Interestingly, **CP 35** assumes a completely different pose, with the thiolate group not involved in zinc coordination and one of the triazole nitrogens hydrogen-bonded to the Lys211 sidechain (Figure 3D). Once again, π-stacking is crucial for the inhibitor to bind the NDM-1 pocket and involves Trp93 and His250, stabilizing **CP 35** triazole and 3-bromophenyl moieties, respectively. The bromine atom of **CP 35** can form a hydrogen bond with a water molecule that in turn interacts via hydrogen bonding with Gln123 peptide nitrogen. 

In this complex, the L3 loop of NDM-1 assumes a slightly more “open” conformation like the complex with **CP 57**, as mentioned above.

### 2.6. Assessment and Refinement of Docking Results

**Figure 4:**
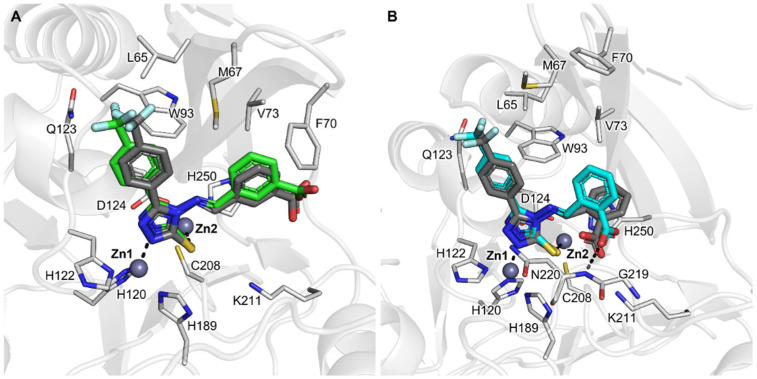
Superpositions of self-docking and crystallographic poses, taken from PDB 8B1Z, and 8B20, of compounds **CP 56** and **CP 57** in NDM-1. (**A**) **CP 56** (docking pose in green sticks); (**B**) **CP 57** (docking pose in cyan sticks). Crystallographic poses are shown in dark grey sticks showing a similar orientation to docking poses (RMSD < 2 Å). The protein is shown in cartoons; the ligand and the residues lining the pocket are displayed in capped sticks. Hydrogen and coordination bonds are shown as dashed black lines.

**Figure 5:**
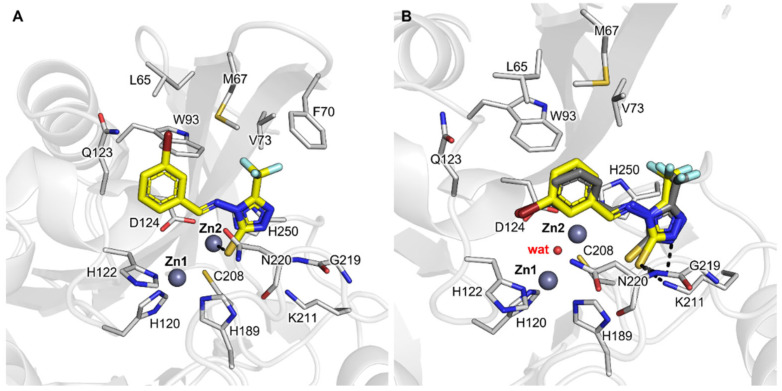
Docking and crystallographic poses of **CP 35** in NDM-1. (**A**) Docking without the catalytic water (PDB 6TGD was used as receptor); (**B**) Self-docking pose of **CP 35** (yellow sticks) retaining catalytic water in the new obtained X-ray structure, in comparison with the crystallographic pose (dark grey sticks; PDB 8B1W). The protein is shown in cartoons; the ligand and the residues lining the pocket are displayed in capped sticks. Hydrogen and coordination bonds are shown as dashed black lines.

As just mentioned, the X-ray structure of NDM-1 in complex with compound **CP 35** returned a binding orientation unpredicted in silico, differently from **CP 56** and **CP 57**, whose crystallographic pose resulted in being very well overlaid to the one assumed in docking simulations in PDB 6TGD (Figure 2, Figure 4 and Figure 5). Indeed, while docking simulations reported **CP 35** coordinating Zn2 by means of the sulfur atom (Figure 5A), X-ray diffraction showed the same sulfur and the nitrogen at the 2-position H-bonded to Asn220 and Lys211 (Figure 5B), respectively, while the trifluoromethyl and bromo-benzylidene moieties formed hydrophobic interactions with L3 residues. Most importantly, the catalytic water molecule located in the middle of the zinc ions was retained in the complex with **CP 35**, differently from the other complexes. We thus performed a self-docking of **CP 35**, retaining the catalytic water, obtaining a much more similar orientation, with an RMSD < 2 Å (Figure 5B). Based on these observations, we could hypothesize a possible alternative binding mode for smaller and more hydrophobic 1,2,4-triazole-thione/thiol-based ligands (e.g., without (4-CF_3_)-phenyl substituent and negatively charged group on the benzylidene moiety in our series), for which the catalytic water can be retained in the binding pocket. A similar result was previously reported for VIM-2 complexed with a 4-methyl-5-(trifluoromethyl)-1,2,4-triazole-3-thiol fragment by Christopeit et al. [23], who observed in the electronic density map a 0.22 and 0.32 occupancy (for A and B chains, respectively) for the hydroxide ion bridging the two zinc ions. In the future, further higher-theory-level simulations may clarify which could be the determinants of 1,2,4-triazole-3-thione/thiol-based compounds for having a certain binding mode in MBLs.

### 2.7. In Vitro Antibacterial Synergistic Activity

The potential synergistic activity of the three best inhibitors (**CP 35**, **CP 56**, and **CP 57**) was tested using the broth microdilution method on six MBL-producing multidrug-resistant clinical isolates [37]. Tested at a fixed concentration of 32 μg/mL (no growth inhibition observed at this concentration when the compound was tested alone), selected compounds were all able to significantly reduce the minimum inhibitory concentration (MIC) of MEM on NDM-1-producing *E. coli* and *K. pneumoniae* bacteria, although limited to a four-fold reduction at best (Table 3). Although apparently modest, this increase in synergistic activity represents a significant improvement over the previous generation of triazole-thione β-lactamase inhibitors previous described by our group [27]. Compound **CP 35** had no synergistic activity at all when tested on VIM-2 *P. aeruginosa* and VIM-4-producing *K. pneumoniae* clinical isolates. Interestingly, a detectable synergistic effect (up to four-fold reduction of MEM MIC) was obtained with both **CP 56** and **CP 57** on VIM-4-producing *K. pneumoniae* (Table 3), although none of the tested compounds showed synergistic activity on *P. aeruginosa*. 

Among tested compounds, **CP 57**, having low micromolar activity toward NDM-1 and VIM-2, gave the best results, reducing meropenem MIC on NDM-1-producing *K. pneumoniae* and VIM-4-producing *K. pneumoniae* by four-fold. Even if tested inhibitors were not able to restore meropenem susceptibility, the obtained results are promising and represent an actual and significant progress over previously designed derivatives, which did not exhibit any significant synergistic activity, although a direct comparison is complicated by the fact that they were tested on different clinical isolates [27]. Our results showed the capability of these new analogues to access, at least partially, the bacterial periplasm and inhibit their enzyme target in a cellular context [21]. 

## 3. Materials and Methods

### 3.1. MIF Analysis for Inhibitor Design and Molecular Docking

Protein selection. The Protein Data Bank (PDB) was checked for MBLs’ structures with co-crystallized competitive inhibitors having a 1,2,4-triazole-3-thione/thiol scaffold similar to that of the tested library. The X-ray structure of NDM-1 complexed with the triazole-based inhibitor OP31 (PDB ID: 6TGD) [24] was selected as a target for the following simulations. 

MIF analysis for inhibitor design. The FLAP software 2.2.2 (Fingerprints for Ligands and Proteins, version 2.2.2.) [38] was used to calculate the binding site Molecular Interaction Fields [39]. Based on the hydrophobic and polar regions resulting from the MIF analysis, several modulations were suggested for the 1,2,4-triazole-3-thione/thiol scaffold to generate a synthetic library to be tested in vitro. 

FlapGlue docking software. FlapGlue is a docking program aimed at detecting favorable binding modes of a ligand within a protein active site using the GRID force field, and can be considered an evolution of GLUE [40]. The source code was completely rewritten in C++ to make it more functional and integrable, retaining the ligand/protein interaction energy calculation method developed and extensively tested first in GRID [41] and later in GLUE. The procedure followed by FlapGlue consists of different steps, some of which are optional. First, a sufficiently large number of conformations of the ligand can be generated, to be considered representative of the entire population. The second step, as optional as the previous one, is to pair the ligand barycenter, or one specific atom, to one or more points within the binding site. These points are either defined externally, or they are distributed using the software to map the pocket uniformly, or they can be selected based on the interaction energy of some GRID probe. In the next step, each conformer is rotated, in a regular manner, about the three cartesian axes having each anchor point as its origin. Then, many orientations are eliminated because of the steric hindrance that occurs whenever the ligand collides with the binding site residues. The preserved orientations represent, although still rather coarse, possible binding modes of the ligand within the site. Each orientation is optimized within the cavity by means of successive torsions and translations. The latter is driven by the ligand–target interaction energy computed by the GRID force field: each small movement is followed by an energy reassessment according to the GRID standard equation (E_GRID_ = E_LJ_ + E_EL_ + E_HB_ + E_ENTROPY_). The final interaction energy between the ligand and the protein binding site is calculated with the following equation: E_FG_ = E_SR_ + E_ES_ + E_HB_ + E_DRY_ (where E_SR_ = steric repulsion energy, E_ES_ = electrostatic energy, E_HB_ = hydrogen bonding energy, E_DRY_ = hydrophobic energy). The final output of the rigid docking of the pregenerated conformational ensemble is a set of solutions ranked according to the corresponding scoring function values, each defined by the 3D coordinates of its atoms. The best poses can be further optimized with a flexible docking procedure, where the conformation and orientation of the ligand can adapt to the site, which instead, in the current version of FlapGlue, remains rigid. Briefly, to the conformation energy calculated according to FlapGlue molecular mechanics, the E_FG_ term is added:

E_MM-FG_ = E_FG_ + E_bond_ + E_angle_ + E_dihedral_ + E_electrostatic_ + E_van der Waals_

All poses selected by rigid docking are then minimized energetically with a Newton conjugation-gradient method.

Molecular docking studies. A semi-rigid docking study was performed using FlapGlue in the 6TGDX-ray structure, in order to evaluate some of the modulations suggested with the MIF analysis. Before running docking simulations, crystallographic waters were removed and the correct protonation and tautomeric states of the zinc coordinating residues were fixed. Considering the chemical nature of the library scaffold and of the binding site, ligand anchor points were determined using a general acceptor FLAP probe (i.e., O probe) and calculating the five lowest energy minima in a 3-Å-radius and 0.25-Å-resolution grid centered on the two zinc ions. Starting from these energy minima points, 25 ligand conformers were generated and roto-translated at 30 different angles for each axis, discarding poses with more than 5% of atoms in steric clash with the protein residues. Then, a number of 1000 and 100 steps were chosen for the following rigid and flexible optimization phases, respectively. Considering the electrostatic effect of zinc ions, lowering the pKa of inhibitors, ligands were modelled in the thiol tautomeric state with the deprotonated sulfur, in agreement with reported evidence [24,25,42,43,44,45]. Previously obtained MBL X-ray models in complex with 1,2,4-triazole-3-thione/thiol-based inhibitors [23,24,25,31] were compared and taken as a reference. We noticed that the coordination between Zn1 and ligand nitrogen 2 and between Zn2 and sulfur was conserved in all structures. Accordingly, the sulfur atom was anchored on the calculated energy minima during all ligand optimization phases. The docking procedure was validated performing a self-docking simulation of compound OP31 in its 6TGD X-ray structure, resulting in good agreement with the crystallographic pose (RMSD heavy = 0.52 Å, Figure S4).

The same procedure was applied for redocking compounds **CP 35**, **CP 56**, and **CP 57** in NDM-1, for which experimental X-ray structures were obtained and are reported in the present paper. In the case of compound **CP 35,** the catalytic water bridging the two zinc ions was not displaced with the inhibitor and, thus, the redocking was performed in the cognate structure described here (PDB ID 8B1W). Considering the presence of the catalytic water, a larger 6-Å-radius grid, centered on the two zinc atoms and on the hydroxide ion, was adopted for calculating energy minima in compound **CP 35** self-docking. 

### 3.2. Chemistry

Chemicals. All chemicals were purchased by Sigma-Aldrich (St. Louis, MO, USA) and used as received. Anhydrous sodium sulfate (Na_2_SO_4_) was used as a drying agent for the organic phases. Organic solvents were removed under reduced pressure at 30 °C. Synthetic-purity solvents were used. 

Instrumentation. Reactions were monitored with thin-layer chromatography on silica-gel-coated aluminum plates (60 Merck F254) and visualized with UV light. ^1^H and ^13^C NMR spectra were recorded on a Jeol ECZ-R 600, at 600 and 150 MHz, respectively, using SiMe_4_ as an internal standard. The following abbreviations are used to designate peak multiplicity: s = singlet, d = doublet, t = triplet, q = quartet, m = multiplet, bs = broad singlet. ESI-MS spectra were recorded on a Micromass Quattro API micro (Waters Corporation, Milford, MA, USA) mass spectrometer. Data were processed using a MassLynx System (Waters). The reverse-phase HPLC analyses that allowed determination of purity of all the compounds were performed with an HP 1100 chromatograph system (Agilent Technologies, Palo Alto, CA, USA) equipped with a quaternary pump (model G1311A), a membrane degasser (G1379A), and a diode-array detector (DAD) (model G1315B) integrated in the HP1100 system. A data analysis was performed using an HP ChemStation system (Agilent Technologies). The analytical column was an Aquasil C18 Thermo Scientific (200 × 4.6 mm, 5 μm particle size) (Waltham, MA, USA). The mobile phase consisted of acetonitrile/water (80/20 *v*/*v*) with 0.1% trifluoroacetic acid and the flow rate was 1.0 mL/min. The injection volume was 20 μL (Rheodyne, Cotati, CA, USA). All compounds were ≥95% pure. 

4-Amino-4H-1,2,4-triazole-3-thiol (2) was synthesized according to study [29]. 

General synthesis of compounds ***CP 17–32.*** To a solution of **2** (0.220 g, 1.89 mmol) in CH_3_COOH (8 mL), the appropriate benzaldehyde was added (1.89 mmol). The reaction mixture was refluxed for 2–4 h, and then was poured into the ice/water mixture. The solid obtained was filtered and crystallized with the solvent reported below. 

(E)-4-((2-(Trifluoromethyl)benzylidene)amino)-4H-1,2,4-triazole-3-thiol **(CP 17).**

Crystallized from Toluene (30% yield). ^1^H-NMR (600 MHz, DMSO-d_6_) δ 14.00 (*s*, 1H), 10.59 (*s*, 1H), 8.93 (*s*, 1H), 8.28 (*d*, J = 6 Hz, 1H), 7.93 (*d*, J = 6 Hz, 1H), 7.88 (*t*, J = 6 Hz, 1H), 7.80 (*t*, J = 6 Hz, 1H). ^13^C-NMR (150 MHz, DMSO-d_6_) δ 162.0, 154.3, 140.4, 133.2, 132.4, 130.0, 128.3 (*q*, ^2^J_F_ = 31.5 Hz), 127.8, 126.4, 123.9 (*q*, ^1^J_F_ = 273 Hz). ESI-MS [M-H]^−^ *m/z* 271.3.

(E)-4-((Naphthalen-1-ylmethylene)amino)-4H-1,2,4-triazole-3-thiol (**CP 18**).

Crystallized from EtOH (27% yield). ^1^H-NMR (600 MHz, DMSO-d_6_) δ 14.00 (*s*, 1H), 10.06 (*s*, 1H), 9.11 (*s*, 1H), 8.91 (*d*, J = 6 Hz, 1H), 8.21 (*d*, J = 6 Hz, 1H), 8.15 (*d*, J = 6 Hz, 1H), 8.09 (*d*, J = 6 Hz, 1H), 7.71 (*m*, 2H), 7.66 (*t*, J = 6 Hz, 1H). ^13^C-NMR (150 MHz, DMSO-d_6_) δ 161.2, 160.9, 153.1, 138.5, 133.5, 131.1, 130.0, 129.4, 128.5, 128.3, 127.2, 126.1, 124.9. ESI-MS [M+H]^+^ *m/z* 255.4. 

(E)-4-((4-Methoxybenzylidene)amino)-4H-1,2,4-triazole-3-thiol **(CP 19).**

Crystallized from EtOH (29% yield). ^1^H-NMR (600 MHz, DMSO-d_6_) δ 8.62 (*s*, 1H), 7.80 (*d*, J = 6 Hz, 2H), 7.27 (*s*, 1H), 6.98 (*d*, J = 6 Hz, 2H), 3.88 (*s*, 3H). ^13^C-NMR (150 MHz, DMSO-d_6_) δ 162.7, 162.6, 161.4, 138.3, 130.6, 124.6, 114.7, 55.6. ESI-MS [M+H]^+^ *m/z* 235.4. 

(E)-4-((3-Methoxybenzylidene)amino)-4H-1,2,4-triazole-3-thiol **(CP 20).**

Crystallized from EtOH (61% yield). ^1^H-NMR (600 MHz, DMSO-d_6_) δ 13.96 (*s*, 1H), 9.45 (*s*, 1H), 8.93 (*s*, 1H), 7.50–7.41 (*m*, 3H), 7.19 (*d*, J = 12 Hz, 1H), 3.83 (*s*, 3H). ^13^C-NMR (150 MHz, DMSO-d_6_) δ 162.8, 161.0, 159.6, 138.3, 133.5, 130.3, 121.4, 118.6, 112.5, 55.3. ESI-MS [M+H]^+^ *m/z* 235.4. 

(E)-4-((2-Methoxybenzylidene)amino)-4H-1,2,4-triazole-3-thiol **(CP 21).**

Crystallized from EtOH (37% yield). ^1^H-NMR (600 MHz, DMSO-d_6_) δ 13.94 (*s*, 1H), 9.87 (*s*, 1H), 8.90 (*s*, 1H), 7.95 (*d*, J = 12 Hz, 1H), 7.60 (*t*, J = 6 Hz, 1H), 7.20 (*d*, J = 12 Hz, 1H), 7.10 (*t*, J = 6 Hz, 1H), 3.34 (*s*, 3H). ^13^C-NMR (150 MHz, DMSO-d_6_) δ 162.3, 159.1, 156.5, 139.1, 134.3, 126.5, 120.9, 120.1, 112.3, 55.9. ESI-MS [M+H]^+^ *m/z* 235.4.

(E)-4-((3-Bromobenzylidene)amino)-4H-1,2,4-triazole-3-thiol **(CP 22).**

Crystallized from EtOH (46% yield). ^1^H-NMR (600 MHz, DMSO-d_6_) δ 13.99 (*s*, 1H), 9.48 (*s*, 1H), 8.84 (*s*, 1H), 8.05 (*s*, 1H), 7.87 (*d*, J = 6 Hz, 1H), 7.81 (*d*, J = 6 Hz, 1H), 7.53 (*t*, J = 6 Hz, 1H). ^13^C-NMR (150 MHz, DMSO-d_6_) δ 163.1, 158.8, 137.9, 134.9, 134.5, 131.4, 130.4, 127.6, 122.3. ESI-MS [M+H]^+^ *m/z* 283.2/285.2.

(E)-4-((2-Bromobenzylidene)amino)-4H-1,2,4-triazole-3-thiol **(CP 23).**

Crystallized from EtOH (53% yield). ^1^H-NMR (600 MHz, DMSO-d_6_) δ 14.04 (*s*, 1H), 10.32 (*s*, 1H), 8.92 (*s*, 1H), 8.10 (*d*, J = 6 Hz, 1H), 7.80 (*d*, J = 6 Hz, 1H), 7.52 (*m*, 2H). ^13^C-NMR (150 MHz, DMSO-d_6_) δ 162.2 158.2, 140.2, 134.9, 133.6, 131.4, 128.4, 128.1, 125.2. ESI-MS [M+H]^+^ *m/z* 283.2/285.2.

(E)-4-((4-Bromobenzylidene)amino)-4H-1,2,4-triazole-3-thiol **(CP 24).**

Crystallized from isopropanol (53% yield). ^1^H-NMR (600 MHz, DMSO-d_6_) δ 13.9 (*s*, 1H), 9.44 (*s*, 1H), 8.94 (*s*, 1H), 7.81 (*m*, 4H). ^13^C-NMR (150 MHz, DMSO-d_6_) δ 163.1, 159.5, 138.0, 132.3, 131.4, 130.3, 126.1. ESI-MS [M+H]^+^ *m/z* 283.5/285.2.

(E)-4-((2-Nitrobenzylidene)amino)-4H-1,2,4-triazole-3-thiol **(CP 25).**

Crystallized from EtOH (89% yield). ^1^H-NMR (600 MHz, DMSO-d_6_) δ 14.05 (*s*, 1H), 10.16 (*m*, 1H), 8.94 (*m*, 1H), 8.21 (*t*, J = 6 Hz, 1H), 8.10 (*t*, J = 6 Hz, 1H), 7.93–7.91 (*m*, 1H), 7.87–7.84 (*m*, 1H). ^13^C-NMR (150 MHz, DMSO-d_6_) δ 163.0, 155.4, 148.6, 138.7, 134.2, 132.8, 129.1, 126.9, 124.9. ESI-MS [M-H]^−^ *m/z* 248.3.

(E)-4-((3-Nitrobenzylidene)amino)-4H-1,2,4-triazole-3-thiol **(CP 26).**

Crystallized from EtOH (53% yield). ^1^H-NMR (600 MHz, DMSO-d_6_) δ 14.02 (*s*, 1H), 9.66 (*d*, J = 3.5 Hz, 1H), 8.99 (*d*, J = 5.8 Hz, 1H), 8.67–8.65 (*m*, 1H), 8.47–8.42 (*m*, 1H), 8.30–8.28 (*m*, 1H), 7.88–7.85 (*m*, 1H). ^13^C-NMR (150 MHz, DMSO-d_6_) δ 163.3, 157.9, 148.2, 137.8, 134.5, 133.9, 130.9, 126.5, 122.5. ESI-MS [M+H]^+^ *m/z* 250.3.

(E)-4-((4-Nitrobenzylidene)amino)-4H-1,2,4-triazole-3-thiol **(CP 27).**

Crystallized from EtOH (85% yield). ^1^H-NMR (600 MHz, DMSO-d_6_) δ 14.02 (*s*, 1H), 9.62 (*m*, 1H), 8.98 (*m*, 1H), 8.39 (*m*, 2H), 8.11 (*m*, 2H). ^13^C-NMR (150 MHz, DMSO-d_6_) δ 163.4, 157.3, 149.2, 138.1, 137.7, 129.5, 124.2. ESI-MS [M+H]^+^ *m/z* 248.3.

(E)-4-((2-Fluorobenzylidene)amino)-4H-1,2,4-triazole-3-thiol **(CP 28).**

Crystallized from EtOH (49% yield). ^1^H-NMR (600 MHz, DMSO-d_6_) δ 13.99 (*s*, 1H), 9.86 (*s*, 1H), 8.99 (*s*, 1H), 8.12–7.91 (*m*, 1H), 7.75–7.59 (*m*, 1H), 7.51–7.26 (*m*, 2H). ^13^C-NMR (150 MHz, DMSO-d_6_) δ 162.7, 160.8, 153.3, 138.7, 134.7, 127.4, 125.2, 120.0 (*d*, ^2^J_F_ = 9.5 Hz), 116.5 (*d*, ^1^J_F_ = 20.2 Hz). ESI-MS [M+H]^+^ *m/z* 223.4. 

(E)-2-(((3-Mercapto-4H-1,2,4-triazol-4-yl)imino)methyl)benzoic acid **(CP 29).**

Crystallized from EtOH (62% yield). ^1^H-NMR (600 MHz, DMSO-d_6_) δ 14.00 (*s*, 1H), 13.60 (*s*, 1H), 10.22 (*s*, 1H), 8.92 (*s*, 1H), 8.03 (*m*, 1H), 8.02 (*m*, 1H), 7.76 (*m*, 1H), 7.70 (*m*, 1H). ^13^C-NMR (150 MHz, DMSO-d_6_) δ 167.5, 162.8, 160.2, 138.7, 132.6, 132.5, 131.9, 131.8, 130.6, 127.9. ESI-MS [M-H]^−^ *m/z* 247.3.

(E)-4-(((3-Mercapto-4H-1,2,4-triazol-4-yl)imino)methyl)benzoic acid **(CP 30).**

Crystalized from EtOH (80% yield). ^1^H-NMR (600 MHz, DMSO-d_6_) δ 13.98 (*s*, 1H), 13.27 (*m*, 1H), 9.54 (*m*, 1H), 8.96 (*s*, 1H), 8.10–7.96 (*m*, 4H). ^13^C-NMR (150 MHz, DMSO-d_6_) δ 166.6, 163.2, 159.1, 137.9, 136.0, 133.7, 129.9, 128.5. ESI-MS [M-H]^−^ *m/z* 247.3. 

(E)-2-(((3-Mercapto-4H-1,2,4-triazol-4-yl)imino)methyl)benzonitrile **(CP 31).**

Crystallized from EtOH (62% yield). ^1^H-NMR (600 MHz, DMSO-d_6_) δ 14.0 (*s*, 1H), 10.26 (*s*, 1H), 8.96 (*s*, 1H), 8.20 (*d*, J = 6 Hz, 1H), 8.03 (*d*, J = 6 Hz, 1H), 7.90 (*t*, J = 6 Hz, 1H), 7.79 (*t*, J = 6Hz, 1H). ^13^C-NMR (150 MHz, DMSO-d_6_) δ 162.4, 155.4, 139.6, 134.5, 134.1, 133.8, 132.6, 127.4, 116.6, 112.1. ESI-MS [M-H]^−^ *m/z* 228.3.

(E)-4-((Pyridin-2-ylmethylene)amino)-4H-1,2,4-triazole-3-thiol **(CP 32).**

A 32% yield. ^1^H-NMR (600 MHz, DMSO-d_6_) δ 14.00 (*s*, 1H), 9.63 (*s*, 1H), 9.04 (*s*, 1H), 8.75 (*m*, 1H), 8.10 (*m*, 1H), 8.00 (*m*, 1H), 7.58 (*m*, 1H). ^13^C-NMR (150 MHz, DMSO-d_6_) δ 163.2, 158.9, 151.3, 150.1, 138.4, 137.4, 126.3, 121.6. ESI-MS [M+H]^+^ *m/z* 206.5, MS [M+Na]^+^ *m/z* 228.4.

4-Amino-5-(trifluoromethyl)-4H-1,2,4-triazole-3-thiol (3) was synthesized according to study [30]. 

General synthesis of compounds **CP 35**, **CP 44**, **CP 45**, and **CP 46.** To a solution of **3** (0.200 g, 1.08 mmol) in CH_3_COOH (6 mL), the appropriate benzaldehyde was added (1.13 mmol). The reaction mixture was refluxed for 2–4 h, and then was poured into the ice/water mixture. The solid obtained was filtered and, when required, purified with the conditions reported below. 

(E)-4-((3-Bromobenzylidene)amino)-5-(trifluoromethyl)-4H-1,2,4-triazole-3-thiol (**CP 35**).

A 56% yield. ^1^H-NMR (600 MHz, DMSO-d_6_) δ 14.94 (*bs*, 1H), 10.07 (*s*, 1H), 8.07 (*s*, 1H), 7.91 (*d*, J = 6 Hz, 1H), 7.86 (*d*, J = 6 Hz, 1H), 7.55–7.57 (*m*, 1H). ^13^C-NMR (150 MHz, DMSO-d_6_) δ 164.0, 163.6, 138.5 (*q*, ^2^J_F_ = 28.5 Hz), 135.9, 133.8, 131.7, 130.9, 127.8, 125.6 (*q*, ^1^J_F_ = 271.5 Hz), 122.5. ESI-MS [M+H]^+^ *m/z* 349.4/351.3.

(E)-3-(((3-Mercapto-5-(trifluoromethyl)-4H-1,2,4-triazol-4-yl)imino)methyl)benzoic acid (**CP 44**).

A 53% yield. ^1^H-NMR (600 MHz, DMSO-d_6_) δ 13.36 (*bs*, 1H), 10.12 (*s*, 1H), 8.42 (*s*, 1H), 8.18 (*d*, J = 7.8 Hz, 1H), 8.13 (*d*, J = 7.8 Hz, 1H), 7.72 (*t*, J = 7.8 Hz, 1H). ^13^C-NMR (150 MHz, DMSO-d_6_) δ 166.4, 164.8, 164.0, 138.7 (*q*, ^2^J_F_ = 40.5 Hz), 133.7, 132.8, 131.9, 131.8, 129.9, 129.2, 116.8 (*q*, ^1^J_F_ = 270 Hz). ESI-MS [M-H]^−^ *m/z* 315.3. 

(E)-2-(((3-Mercapto-5-(trifluoromethyl)-4H-1,2,4-triazol-4-yl)imino)methyl)benzoic acid (**CP 45**).

Purified with flash chromatography using CHCl_3_/HCOOH 0.1% as the eluent (48% yield). ^1^H-NMR (600 MHz, DMSO-d_6_) δ 10.91 (*s*, 1H), 8.05 (*d*, J = 6 Hz, 1H), 8.00 (*d*, J = 6 Hz, 1H), 7.72–7.78 (*m*, 2H). ^13^C-NMR (150 MHz, DMSO-d_6_) δ 167.6, 163.9, 162.9, 139.0 (*q*, ^2^J_F_ = 40.5 Hz), 132.6, 132.5, 132.3, 131.7, 130.7, 127.4, 116.8 (*q*, ^1^J_F_ = 270 Hz). ESI-MS [M-H]^−^ *m/z* 315.3.

(E)-4-(((2H-Tetrazol-5-yl)methylene)amino)-5-(trifluoromethyl)-4H-1,2,4-triazole-3-thiol (**CP 46**).

2H-tetrazole-5-carbaldehyde was synthetized according to study [46].

The reaction mixture was extracted with EtOAc (2 × 20 mL), dried over Na_2_SO_4_, and concentrated to dryness. Purification of the residue with flash chromatography using DCM/isopropanol (90/10 *v*/*v*) + 0.1% of HCOOH as the eluent gave the target compound (20% yield). ^1^H-NMR (600 MHz, DMSO-d_6_) δ 10.41 (*bs*, 1H). ^13^C-NMR (150 MHz, DMSO-d_6_) δ 164.0, 154.3, 153.1, 138.8 (*q*, ^2^J_F_ = 40.5 Hz), 116.6 (*q*, ^1^J_F_ = 270 Hz). ESI-MS [M-H]^−^ *m/z* 263.3.

4-(Trifluoromethyl)benzohydrazide (6), Potassium 2-(4-(trifluoromethyl)benzoyl)hydrazine-1-carbodithioateand (7), and 4-Amino-5-(4-(trifluoromethyl)phenyl)-4H-1,2,4-triazole-3-thiol (8) were synthesized according to study [32]. General synthesis of compounds **CP 55-58**. To a solution of **8** (0.260 g, 1.00 mmol) in CH_3_COOH (8 mL), the appropriate benzaldehyde was added (1.05 mmol). The reaction mixture was refluxed for 2–5 h, and then was poured into the ice/water mixture. The solid obtained was filtered and purified with flash chromatography or with crystallization by using the conditions reported below. 

(E)-4-((3-Bromobenzylidene)amino)-5-(4-(trifluoromethyl)phenyl)-4H-1,2,4-triazole-3-thiol (**CP 55**).

Purified with flash chromatography using PE/EtOAc (90/10 *v*/*v*) as the eluent (12% yield). ^1^H-NMR (600 MHz, DMSO-d_6_) δ 14.45 (*bs*, 1H), 9.82 (*s*, 1H), 8.07–8.11 (*m*, 3H), 7.92–7.94 (*m*, 3H), 7.84–7.86 (*m*, 1H), 7.52–7.55 (*m*, 1H). ^13^C-NMR (150 MHz, DMSO-d_6_) δ 165.1, 162.7, 147.5, 135.5, 134.2, 131.5, 131.1, 130.5 (*q*, ^2^J_F_ = 31.5 Hz), 129.2, 129.1, 127.7, 125.7, 123.8 (*q*, ^1^J_F_ = 271.5 Hz), 122.4. ESI-MS [M-H]^−^ *m/z* 425.3/427.3.

(E)-3-(((3-Mercapto-5-(4-(trifluoromethyl)phenyl)-4H-1,2,4-triazol-4-yl)imino)methyl)benzoic acid (**CP 56**).

Purified with flash chromatography using CHCl_3_/HCOOH 0.1% as the eluent (27% yield). ^1^H-NMR (600 MHz, DMSO-d_6_) δ 14.45 (*bs*, 1H), 13.35 (*bs*, 1H), 9.88 (*s*, 1H), 8.39 (*s*, 1H), 8.16–8.19 (*m*, 2H), 8.10 (*d*, J = 8.2 Hz, 2H), 7.92 (*d*, J = 8.2 Hz, 2H), 7.70–7.73 (*m*, 1H). ^13^C-NMR (150 MHz, DMSO-d_6_) δ 166.5, 166.1, 162.7, 147.5, 133.3, 132.5, 132.3, 131.8, 130.6 (*q*, ^2^J_F_ = 31.5 Hz), 129.8, 129.6, 129.3, 129.1, 125.7, 123.9 (*q*, ^1^J_F_ = 271.5 Hz). ESI-MS [M-H]^−^ *m/z* 391.4.

(E)-2-(((3-Mercapto-5-(4-(trifluoromethyl)phenyl)-4H-1,2,4-triazol-4-yl)imino)methyl)benzoic acid (**CP 57**). 

Crystallized from EtOH/H_2_O (43% yield). ^1^H-NMR (600 MHz, DMSO-d_6_) δ 14.43 (*bs*, 1H), 13.62 (*bs*, 1H), 10.62 (*s*, 1H), 8.07–8.11 (*m*, 3H), 7.99–8.02 (*m*, 1H), 7.91 (*d*, J = 8.2 Hz, 2H), 7.71–7,76 (*m*, 2H). ^13^C-NMR (150 MHz, DMSO-d_6_) δ 167.6, 164.9, 162.7, 147.7, 132.5, 132.3, 132.1, 130.6, 130.5 (*q*, ^2^J_F_ = 33 Hz), 129.3, 129.2, 127.8, 125.6, 125.7, 123.8 (*q*, ^1^J_F_ = 271.5 Hz). ESI-MS [M-H]^−^ *m/z* 391.4.

(E)-4-(((2H-Tetrazol-5-yl)methylene)amino)-5-(4-(trifluoromethyl)phenyl)-4H-1,2,4-triazole-3-thiol (**CP 58**).

2H-Tetrazole-5-carbaldehyde was synthesized according to study [47]. 

A 42% yield. ^1^H-NMR (600 MHz, DMSO-d_6_) δ 14.62 (*s*, 1H), 10.51 (*s*, 1H), 8.12 (*d*, J = 12 Hz, 2H), 7.92 (*d*, J = 12 Hz, 2H). ^13^C-NMR (150 MHz, DMSO-d_6_) δ 172.1, 162.6, 150.2, 148.0, 130.7 (*q*, ^2^J_F_ = 31.5 Hz), 129.6, 128.9, 125.7, 123.9 (*q*, ^1^J_F_ = 271.5 Hz). ESI-MS [M-H]^−^ *m/z* 339.1.

### 3.3. In Vitro Enzyme Inhibition and Microbiological Assays

The percentage of inhibition (%Inh) of 1,2,4-triazole derivatives was determined against a panel of subclass B1 MBLs (NDM-1, VIM-1, VIM-2, and IMP-1). Each compound was initially tested at a fixed concentration for inhibitory activity vs. the targeted enzymes. For the best active inhibitors, half-maximal inhibitory concentration (*IC_50_*) and *K_i_* values were determined. Reactions were monitored using a Jasco V-730 spectrophotometer ( JASCO EUROPE S.R.L. Via Luigi Cadorna 1 23894 Cremella (LC) Italy) using nitrocefin or meropenem as reported substrates. Compounds were dissolved in dimethyl sulfoxide (DMSO) and stored at −20 °C. For VIM-1, VIM-2, and IMP-1MBLs, assays were conducted in 20 mM of HEPES, 100 mM of NaCl, and ZnSO_4_ at 10 µM at pH 7.4 at 25 °C with 0.01% *v*/*v* Triton X-100 to avoid compound aggregation and promiscuous inhibition [48]. For NDM-1, assays were conducted in 20 mM of TRIS HCl + 150 mM of NaCl at pH 7.5 at 25°C with 0.01% *v*/*v* Triton X-100 to avoid aggregation. 

Meropenem was used as a reporter substrate (λ= 298 nm) at a concentration of 57 µM for NDM-1 (*K_m_* = 63.4 µM), 17 µM for VIM-2 (*K_m_* = 8.9 µM), and 57 µM for IMP-1 (*K_m_* = 64 µM). For all targeted proteins, the reaction was typically initiated by adding the substrate to the reaction buffer last, after a 5 min preincubation. After brief shaking, the reading was performed for a total kinetic time of 240 s at 25 °C. The *IC_50_* values were determined by measuring the rate of hydrolysis of the reporter substrate in the presence of five different inhibitor concentrations. The binding affinity *K_i_* was generally estimated from the determined *IC_50_* using the Cheng–Prusoff equation as per competitive inhibition. For the best inhibitors, the mechanism of inhibition and *K_i_* were determined with a Dixon plot (**CP 22**, **CP 35**, **CP 56**, and **CP 57** against NDM-1; compound **CP 57** against VIM-2).

The synergistic activity of compounds **CP 35**, **CP 56**, and **CP 57** was evaluated with meropenem on MBL-producing clinical isolates [13] by determining the minimum inhibitory concentration (MIC) of the antibiotic, in triplicate, using cation-supplemented Mueller–Hinton broth and a bacterial inoculum of 5 × 10^4^ CFU/well, as recommended by the CLSI [47] in both the absence and presence of a fixed concentration (32 μg/mL) of the inhibitor, as previously described [25]. As an internal reference quality control, the recently described JMV7061 MBL inhibitor [26] was used and reproducibly provided meropenem MIC values ranging from 0.06 to 0.25 µg/mL with MBL-producing Enterobacterales or 8 µg/mL with *Pseudomonas aeruginosa* VA-182/00.

### 3.4. NDM-1 Overexpression, Purification, and Crystallization in Complex with Inhibitors

Recombinant NDM-1 was overexpressed in *E. coli* BL21(DE3) and purified as previously described [49]. Protein crystallization was carried out using the sitting-drop isothermal vapor diffusion setup. Drops of a 1.2 µL volume were dispensed using an Oryx 8 crystallization robot (Douglas Instruments Ltd., Berkshire, UK) by mixing equal volumes of a 60 mg/mL NDM-1 solution (in 20 mM HEPES, 100 mM NaCl, pH 7.0) and precipitant buffer (0.1 M HEPES, 0.1 M MOPS, pH 7.5, 0.03 M MgCl_2_ × 6H_2_O, 0.03 M CaCl_2_ × 2H_2_O, 12.5% *v*/*v* MPD, 12.5% PEG 1000, 12.5% *w*/*v* PEG 3350) and optimized by microseeding. NDM-1 Crystals grew within a 48 h incubation at 293 K and then were soaked for 24–72 h in 5 mM solutions of **CP 35**, **CP 56**, or **CP 57** inhibitors dissolved in 0.1 M HEPES, 0.1 M MOPS, pH 7.5, 0.05 M NaCl, 12.5% *v*/*v* MPD, 12.5% PEG 1000, 12.5% *w*/*v* PEG 3350, 5% *v*/*v* DMSO. Crystals were cryo-protected with 20% *v*/*v* ethylene glycol before freezing in liquid nitrogen. 

### 3.5. Crystallography-Structure Building and Refinement

X-ray diffraction data were collected at the ID23-1 beamline at the European Synchrotron Radiation Facility (ESRF, Grenoble, France). Space group determination and reflections indexing were carried out with the available automated processing pipelines (GrenADES, EDNA Autoprocessing, and autoPROC). Further data reduction was performed with Aimless via the CCP4i2 interface [50].

v11.0 The initial protein model was obtained with molecular replacement (Molrep software [51]) using PDB 6TGD as a template and then manually adjusted and automatically refined with Coot [52] and Refmac5 [53], respectively. Anomalous maps confirmed the presence of metal atoms (two zinc ions in the catalytic pocket of NDM-1 and one calcium ion in a peripheral site in each protein chain) while inhibitors were detected with difference maps and stereochemically modelled with AceDRG [54]. Omit maps at 2.5 σ were calculated by Phenix v1.20.1 [55] to assess the presence of the inhibitors. Interactions of inhibitors with NDM-1 were analyzed with PLIP (https://plip-tool.biotec.tu-dresden.de/plip-web/plip/index, accessed on 28 August 2023) [56]. 

## 4. Conclusions

A new series of 24 derivatives was designed in silico, synthesized, and tested in vitro against purified NDM-1 and in microbiological assays against MBL-producing multidrug-resistant clinical isolates.

The introduced chemical modifications yielded compounds with low micromolar inhibitory activity on NDM-1, but also on VIM-type MBLs. The most active compound in the library turned out to be the carboxylate derivative CP-57, for which the introduction of a 4-trifluoromethylphenyl substituent in position 5 of the 1,2,4-triazole-3-thiol core led to a remarkable improvement in activity with respect to its simpler analogue, also in terms of potentiation of meropenem, a carbapenem antibiotic. However, it should be considered that the activity of these compounds still requires further optimization. Indeed, other inhibitors active on MBLs (such as taniborbactam or QPX7728), as well as compounds showing structural similarity (i.e., the 1,2,4-triazole-3-thione JMV7061 [26]), show a much higher inhibitory potency (*Ki* values in the low nanomolar range) and synergistic activity (i.e., significantly below the resistance breakpoint) [26,57,58].

Nonetheless, X-ray-crystallography studies provided interesting insights on the role played by the designed chemical modifications introduced in this novel series of compounds in the modulation of potency. Interestingly, and unlike previously developed analogues, the modifications introduced in the best compounds showed, besides an improvement in their inhibitory activity, an apparently broadened spectrum of activity, as reflected by biochemical data and the detectable synergistic activity on NDM-1- and VIM-4-producing *Enterobacterales* clinical isolates. Compound CP-57 and analogues are currently undergoing further investigations to evaluate their potency toward other clinically relevant MBL variants in vitro and in vivo.

## Figures and Tables

**Table 1:** The inhibitory activity of 1,2,4-triazole-3-thiol derivative analogues vs. NDM-1.

Compound	Structure	NDM-1 *IC*_50_ (μM)
**CP 17**	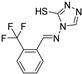	226 ± 26
**CP 18**	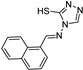	52 ± 7.5
**CP 22**	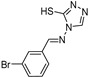	88.5 ± 3.7 *(K_i_* of 42.7 ^a^ ± 2.1)
**CP 23**	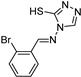	83.0 ± 3.3
**CP 24**	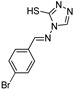	83.1 ± 2.7
**CP 29**	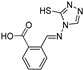	NI ^b^
**CP 30**	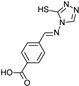	NI ^b^

Kinetics were monitored at 25 °C by following the absorbance variation observed upon substrate MEM hydrolysis. Assays were performed in triplicate. ^a^ K_i_ was determined with Dixon Plots. ^b^ NI: no inhibition (<30% inhibition at 100 µM).

**Table 2:** The inhibitory activity of 4-amino-5-(trifluoromethyl)-4H-1,2,4-triazole-3-thiol and 4-amino-5-(4-(trifluoromethyl)phenyl)-4H-1,2,4-triazole-3-thiol derivatives against NDM-1 and VIM-2 MBLs.

Compound	Structure	NDM-1 *K_i_* (μM)	VIM-2 *K_i_* (μM)
**CP 35**	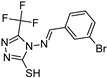	25.8 ^a^ ± 0.7	22.6 ^b^ ± 1.1
**CP 44**	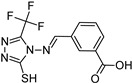	115 ^b^ ± 20	22.3 ^b^ ±1.1
**CP 45**	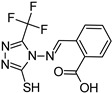	83 ^b^ ± 4.1	NI
**CP 46**	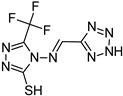	70 ^b^ ± 3.2	215 ^b^ ± 10
**CP 55**	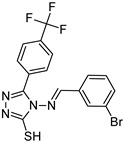	220 ^b^ ± 11	40 ^b^ ± 2
**CP 56**	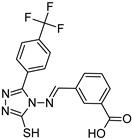	24.5 ± 0.5	13.8 ^b^ ± 1.6
**CP 57**	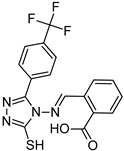	5.6 ± 0.8	4.1 ± 0.8
**CP 58**	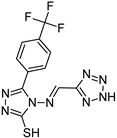	78 ^b^ ± 8	28 ^b^ ± 3.2

Kinetics were monitored at 25 °C by following substrate MEM hydrolysis. Assays were performed in triplicate. NI: no inhibition = < 30% inhibition at 100 µM. ^a^ K_i_ was determined with Dixon Plots; ^b^ K_i_ was calculated from IC_50_ as per competitive inhibition [32].

**Table 3:** Antibacterial synergistic activity of compounds on NDM-1-, VIM-2-, and VIM-4-producing clinical isolates with MEM determined using the broth microdilution method.

Cpd (32 μg/mL)		MEM MIC (μg/mL)					
		*E. coli* MO-287 (NDM-1)	*E. coli* MO-978 (NDM1)	*K.* *pneumoniae* SI-1072 (NDM-1)	*K.* *pneumoniae* SI-518 (NDM-1)	*K.* *pneumoniae* VA-416/02 (VIM-4)	*P.* *aeruginosa* VA-182/00 (VIM-2)
None		64	256	128	128	16	256
**CP 35**	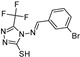	64	128	32	32	16	256
**CP 56**	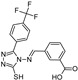	32	256	32	64	8	256
**CP 57**	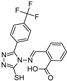	32	256	32	32	4	256

## Data Availability

Data is contained within the article and Supplementary Material.

## Supplementary Materials

The following supporting information can be downloaded at: https://www.mdpi.com/article/10.3390/ph16121682/s1, Table S1: Structures of compounds and corresponding aldehydes used; Table S2: The inhibitory activity of 4-amino-4H-1,2,4-triazole-3-thiol derivatives discussed here; Figure S1: Dixon plots for compounds CP 35 and CP 56 against NDM-1 and CP 57 against NDM-1 and VIM-2; Figure S2: ^1^H NMR spectra of the reported final compounds; Table S3: X-ray crystallographic data for NDM-1 in complex with CP 35, CP 56, and C P57; Figure S3: Structure–activity relationship for synthesized compounds; Figure S4: Validation of molecular docking protocol.
