# Supplementary material for: Structure-Based Optimization of 1,2,4-Triazole-3-Thione Derivatives: Improving Inhibition of NDM-/VIM-Type Metallo-β-Lactamases and Synergistic Activity on Resistant Bacteria
Source: Pharmaceuticals (Basel). 2023 Dec 2;16(12):1682. doi: 10.3390/ph16121682 (PMC10747250; doi:10.3390/ph16121682)

Structure-Based Optimization of 1,2,4-Triazole-3-Thione Derivatives: Improving Inhibition of NDM-/VIM-Type Metallo-β-Lactamases and Synergistic Activity on Resistant Bacteria

Matteo Bersani [1,†], Mariacristina Failla [1,†], Filippo Vascon [2,†], Eleonora Gianquinto [1], Laura Bertarini [3], Massimo Baroni [4], Gabriele Cruciani [5], Federica Verdirosa [6], Filomena Sannio [6], Jean-Denis Docquier [6,7], Laura Cendron [2], Francesca Spyrakis [1], Loretta Lazzarato [1,*] and Donatella Tondi [3,*]

[1] Department of Drug Science and Technology, University of Turin, Via Pietro Giuria 9, 10125 Turin, Italy; matteo.bersani@unito.it (M.B.); mariacristina.failla@unito.it (M.F.); eleonora.gianquinto@unito.it (E.G.); francesca.spyrakis@unito.it (F.S.)
[2] Department of Biology, University of Padua, Viale G. Colombo 3, 35121 Padua, Italy; filippo.vascon@unipd.it (F.V.); laura.cendron@unipd.it (L.C.)
[3] Department of Life Sciences, University of Modena and Reggio Emilia, Via Campi 103, 41125 Modena, Italy; laura.bertarini@unimore.it
[4] Kinetic Business Centre, Molecular Discovery Ltd., Elstree, Borehamwood, Hertfordshire WD6 4PJ, UK; massimo@moldiscovery.com
[5] Department of Chemistry, Biology and Biotechnology, Università Degli Studi di Perugia, Via Elce di Sotto, 06132 Perugia, Italy; gabriele.cruciani@unipg.it
[6] Department of Medical Biotechnologies, University of Siena, Viale Bracci 16, 53100 Siena, Italy; federica.verdirosa95@gmail.com (F.V.); filomena.sannio@unisi.it (F.S.); jddocquier@unisi.it (J.-D.D.)
[7] Laboratoire de Bactériologie Moléculaire, Centre d'Ingénierie des Protéines-InBioS, Université de Liège,
B-4000 Liège, Belgium
* Correspondence: loretta.lazzarato@unito.it (L.L.); donatella.tondi@unimore.it (D.T.)
† These authors contributed equally to this work.

Table S1. Structures of compounds and corresponding aldehydes used.

Cpd	Structure	Aldehyde	Cpd	Structure	Aldehyde
**CP 17**	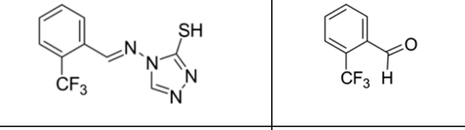		**CP 29**	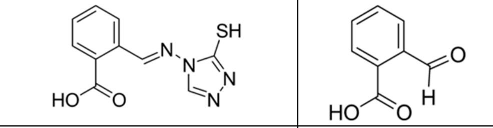	
**CP 18**	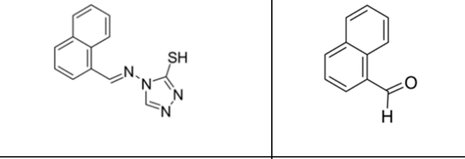		**CP 30**	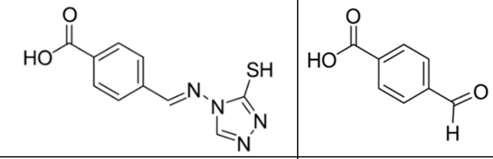	
**CP 19**	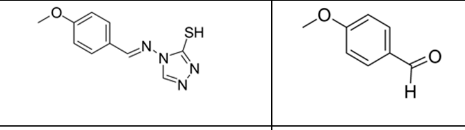		**CP 31**	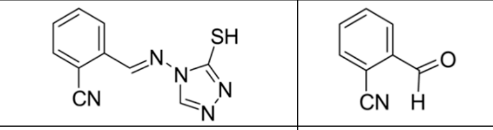	
**CP 20**	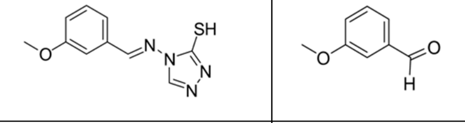		**CP 32**	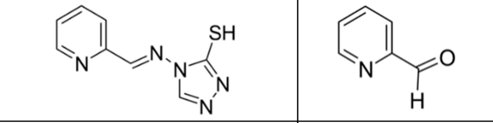	
**CP 21**	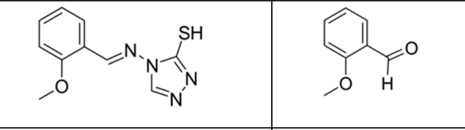		**CP 35**	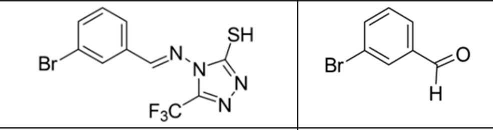	
**CP 22**	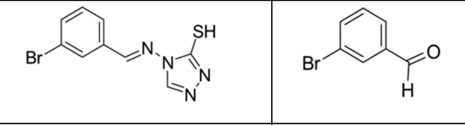		**CP 44**	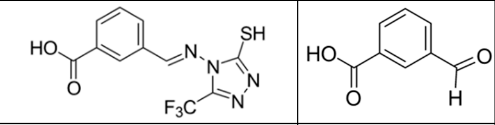	
**CP 23**	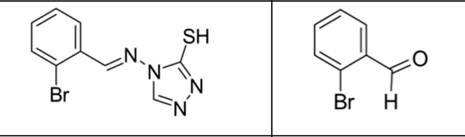		**CP 45**	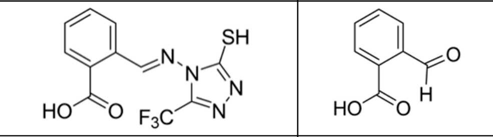	
**CP 24**	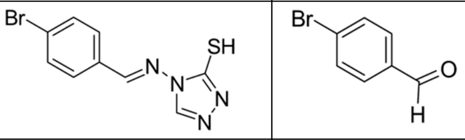		**CP 46**	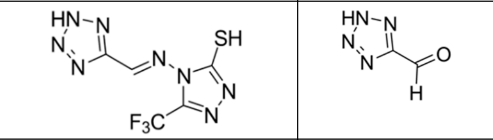	
**CP 25**	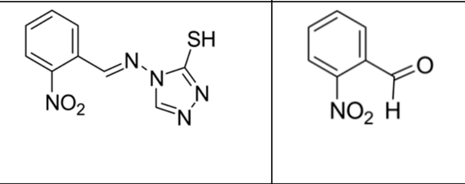		**CP 55**	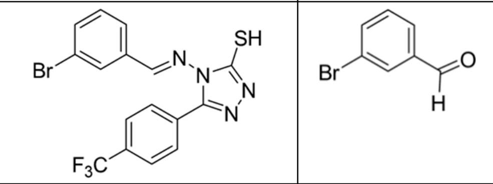	
**CP 26**	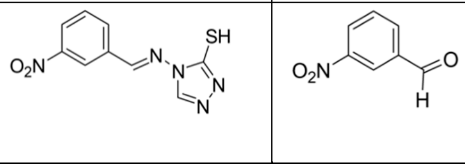		**CP 56**	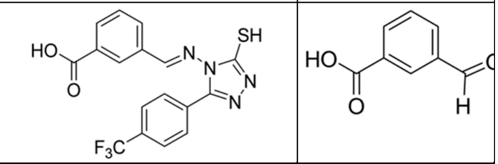	
**CP 27**	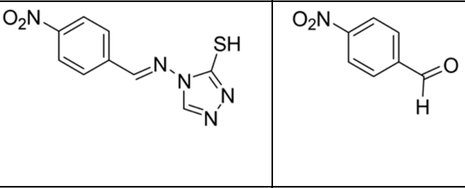		**CP 57**	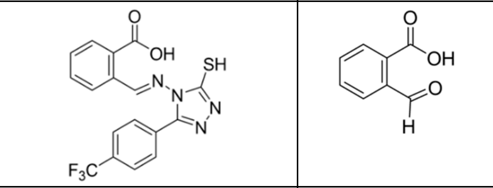	
**CP 28**	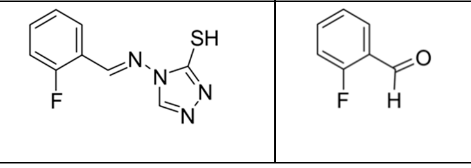		**CP 58**	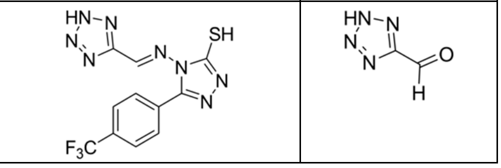	

Table S2. The inhibitory activity of 4-amino-4H-1,2,4-triazole-3-thiol derivatives discussed here.

CODE	% Inib *vs* VIM[a,b] MBLs	NDM-1
**CP 17**	46[a]	51
**CP 18**	33[a]	>100
**CP 19**	15[a]	24
**CP 20**	14[a]	40
**CP 21**	32[a]	38
**CP 22**	46[a]	68
**CP 23**	NI	67
**CP 24**	28[a]	64
**CP 25**	23[a]	11
**CP 26**	NI	NI
**CP 27**	NI	36
**CP 28**	NI	NI
**CP 29**	17[a]	NI
**CP 30**	18[a]	NI
**CP 31**	NI	10
**CP 32**	17[a]	20
**CP 35**	53 [a, b]	81
**CP 44**	42 [a]<br>47[b]	24
**CP 45**	16 [a]	20
**CP 46**	42[b]	15
**CP 55**	48 [a]<br>75[b]	NI
**CP 56**	77 [a]<br>42[b]	>100
**CP 57**	73 [a]<br>>100[b]	>100
**CP 58**	28 [a]<br>53[b]	52

Tests were conducted at 25° C by following the absorbance variation observed upon reporter substrate (Meropenem 57 µM for NDM-1, 17 µM for VIM-2) or Nitrocefin (23 µM for VIM-1). The percentage of inhibition is reported except when ≤ 15% and in this case is indicated by NI "no inhibition". Compounds concentration was [a]200 mM *vs* VIM-1, [b] 70 mM *vs* VIM-2 and 100 mM *vs* NDM-1. Assays were run after a 5-minutes incubation.

Figure S1. Dixon plots for compounds **CP 35**, **CP 56** against NDM-1(**A,B**) and **CP 57** against NDM-1 and VIM-2 (**C,D**). Compounds were tested after a 5-minutes incubation, using MEM as reporter substrate. The initial velocity of the reaction is measured as a function of inhibitor concentration at three fixed concentrations of MEM. The data where then plotted as 1/V as a function of [I] for each substrate concentration, and the value of K_i determined from the |x-axis| value at which the lines intersect. Data points are mean ± SD obtained from two or three independent experiments.

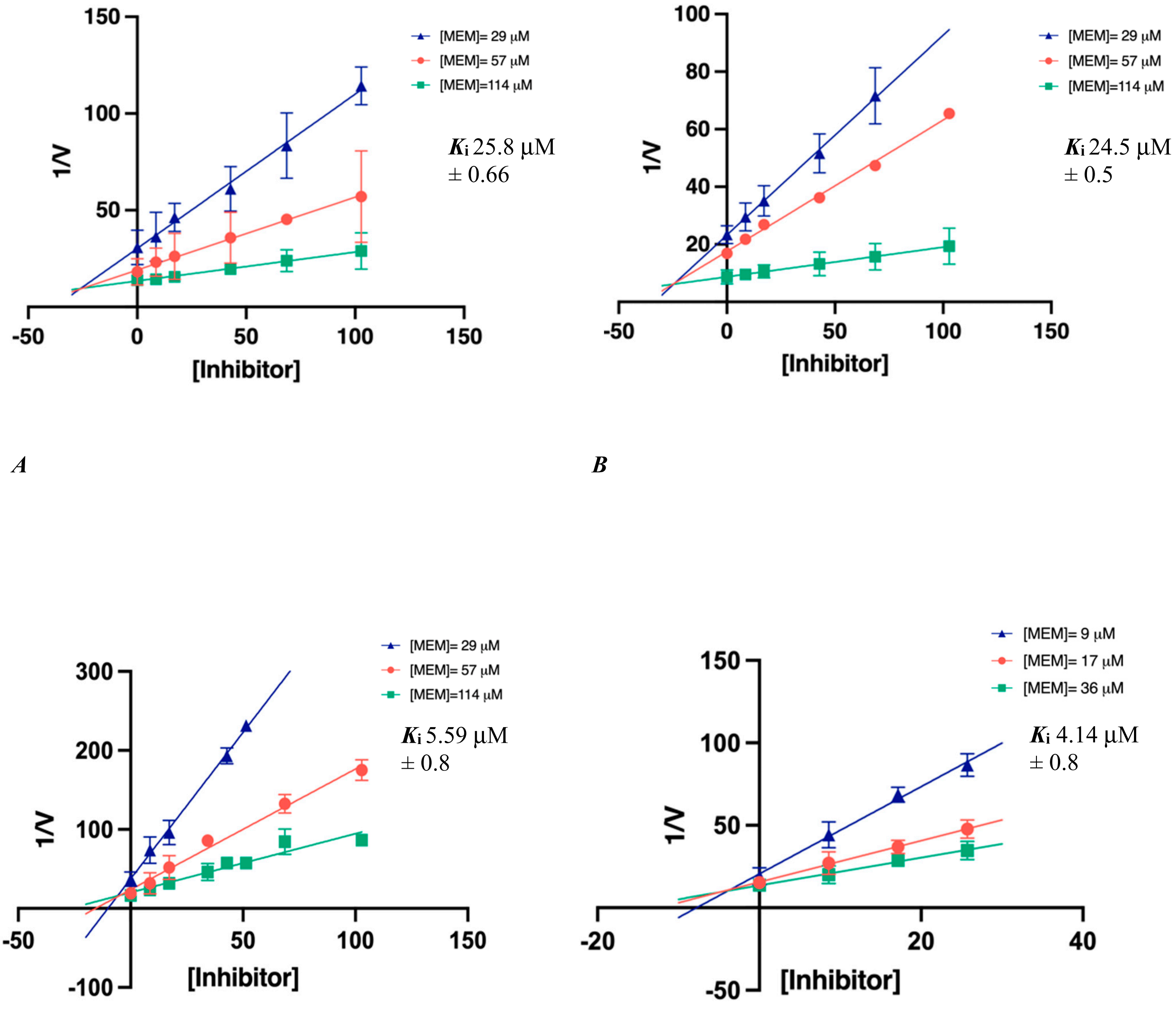

Table S3. X-Ray crystallographic data for NDM-1 in complex with **CP 35**, **CP 56** and **CP P57**.

	**NDM-1/CP 35**	**NDM-1/CP 56**	**NDM-1/CP 57**
PDB ID	8B1W	8B1Z	8B20
**Data collection statistics**			
Diffraction source	ID23-1 (ESRF)	ID23-1 (ESRF)	ID23-1 (ESRF)
Wavelength (Å)	0.873	0.873	0.873
Temperature (K)	100	100	100
Detector	Eiger2 XE 16M	Eiger2 XE 16M	Eiger2 XE 16M
Crystal-detector distance (mm)	167.1	144.9	167.1
Rotation range per image (°)	0.15	0.10	0.10
Exposure time per image (s)	0.013	0.016	0.019
Space group	$P2_12_12_1$	$P2_12_12_1$	$P2_12_12_1$
No. of molecules/ASU	2	2	2
a, b, c (Å)	70.19 73.70 77.28	68.61 73.96 77.11	77.07 70.44 73.8
Total no. of reflections	241383 (10302)	345446 (16227)	277691 (15290)
No. of unique reflections	44671 (2328)	52482 (2570)	39230 (2195)
Completeness (%)	99.7 (99.5)	100 (100)	100 (100)
Redundancy	5.4 (4.4)	6.6 (6.3)	7.1 (7.0)
$\langle I/\sigma(I)\rangle$	8.6 (2.2)	6.8 (2.1)	6.8 (2.4)
R_{mrg}	0.194 (0.531)	0.161 (0.920)	0.178 (0.956)
Overall B factor from Wilson plot (Å^2)	19.1	19.7	22.3
**Refinement statistics**			
Resolution range (Å)	36.88 – 1.70	34.19 – 1.60	53.31 – 1.78
No. of reflections, working set	44139	52418	39169
No. of reflections, test set	2185	2595	1900
Final R_{cryst}	0.192	0.174	0.184
Final R_{free}	0.222	0.209	0.207
No. of non-H atoms<br>Protein<br>Zn ions<br>Inhibitor<br>Others<br>Water<br>Total	 3442<br>4<br>38<br>31<br>153<br>3668	 3452<br>4<br>54<br>36<br>189<br>3735	 3442<br>4<br>27<br>30<br>134<br>3637
R.m.s. deviations<br>Bonds (Å)<br>Angles (°)	 0.009<br>1.568	 0.009<br>1.642	 0.008<br>1.574
Average B factors (Å^2)	22.0	23.0	26.0
Ramachandran plot<br>Most favored (%)<br>Allowed (%)	 98<br>2	 98<br>1	 98<br>2
RSCC inhibitor (Chain ID)	0.94 (A) ; 0.90 (B)	0.93 (A) ; 0.94 (B)	0.91 (A)

Figure S2. ^{1}H NMR spectra of the reported final compounds (CP17-CP32, CP35, CP44-CP46, CP55-CP58).

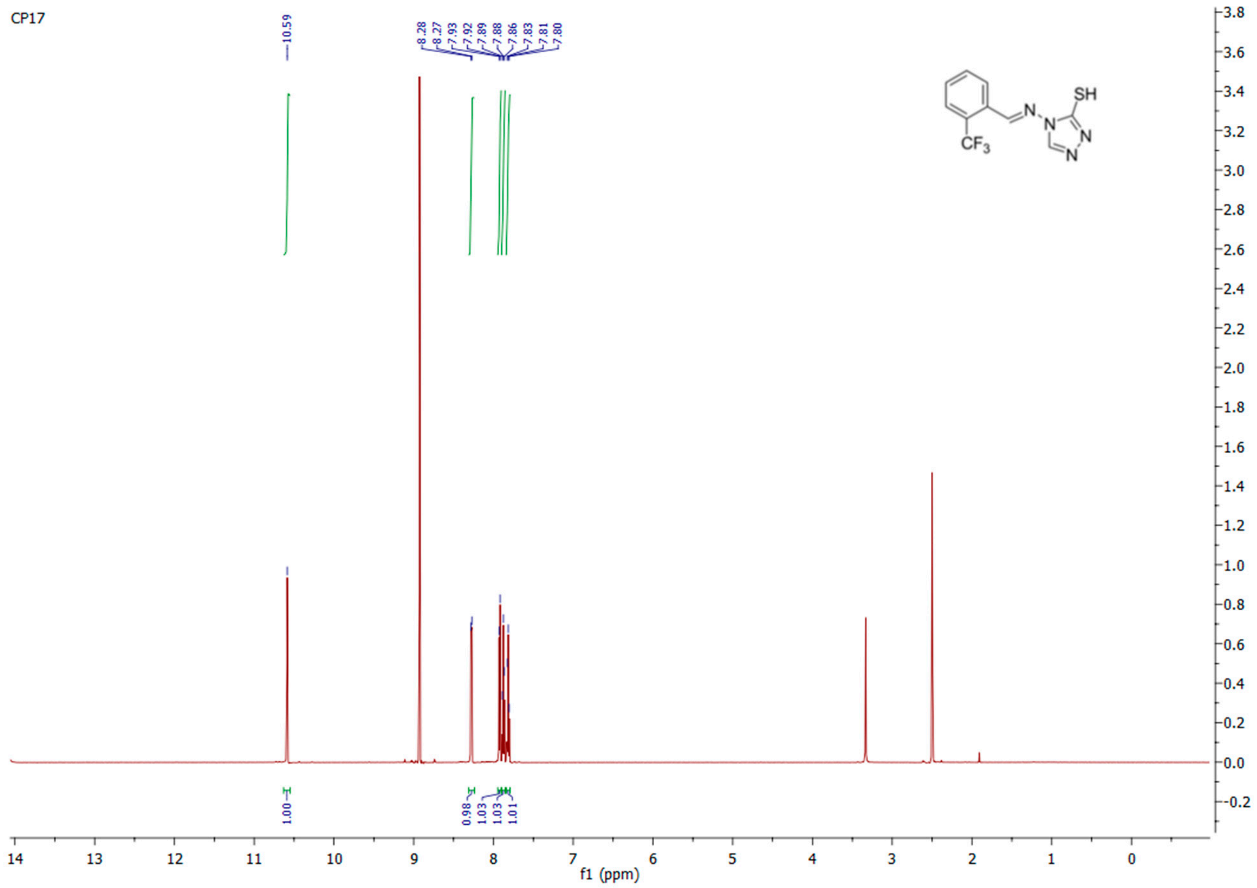

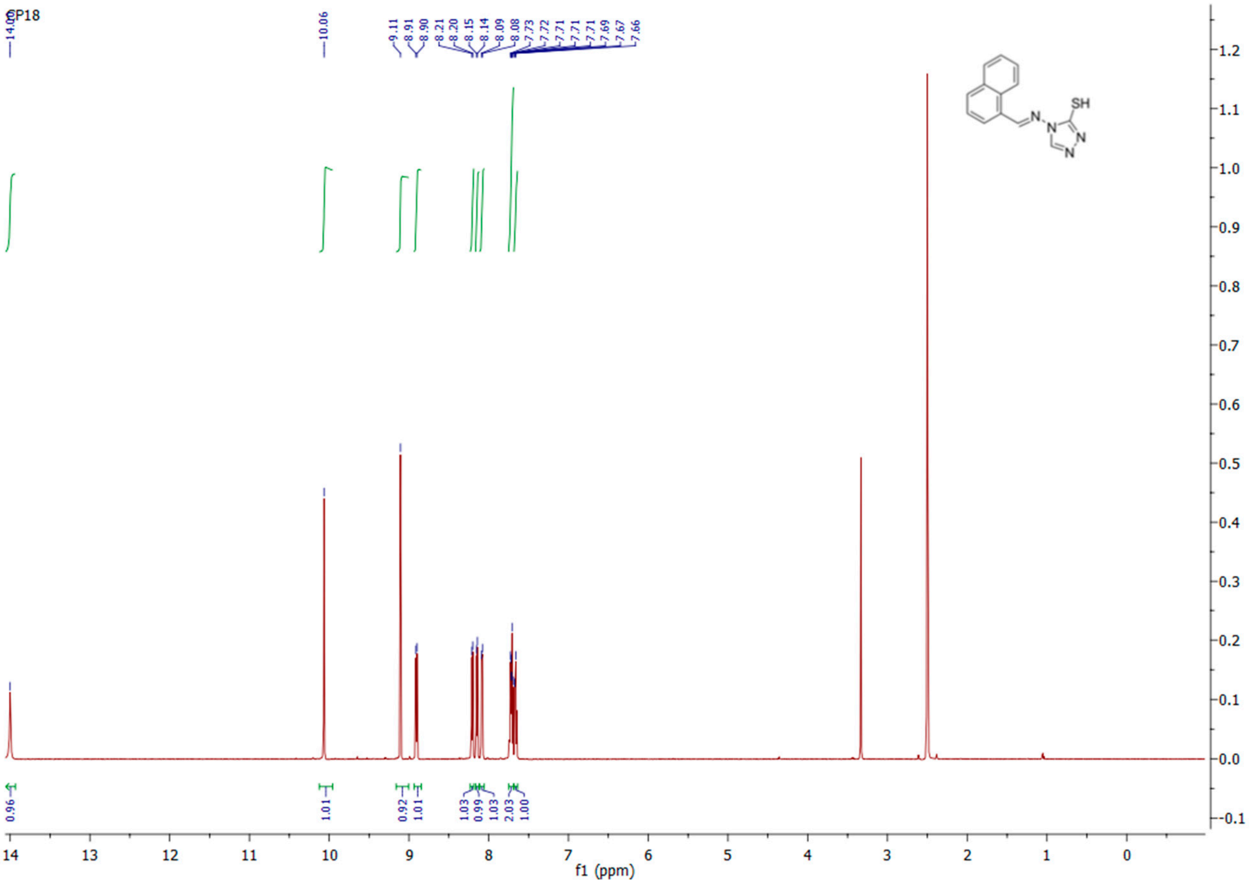
CP18
f1 (ppm)

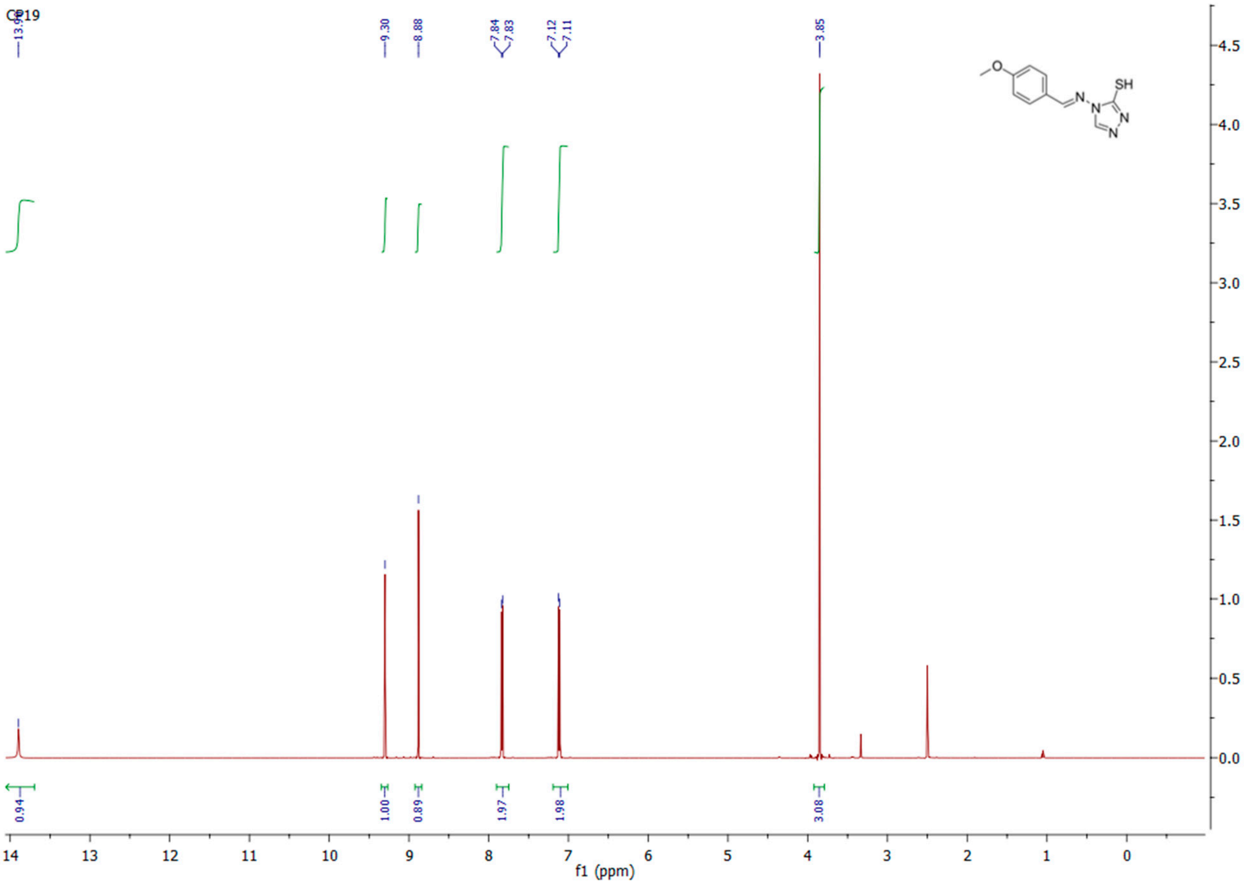
CP19
f1 (ppm)

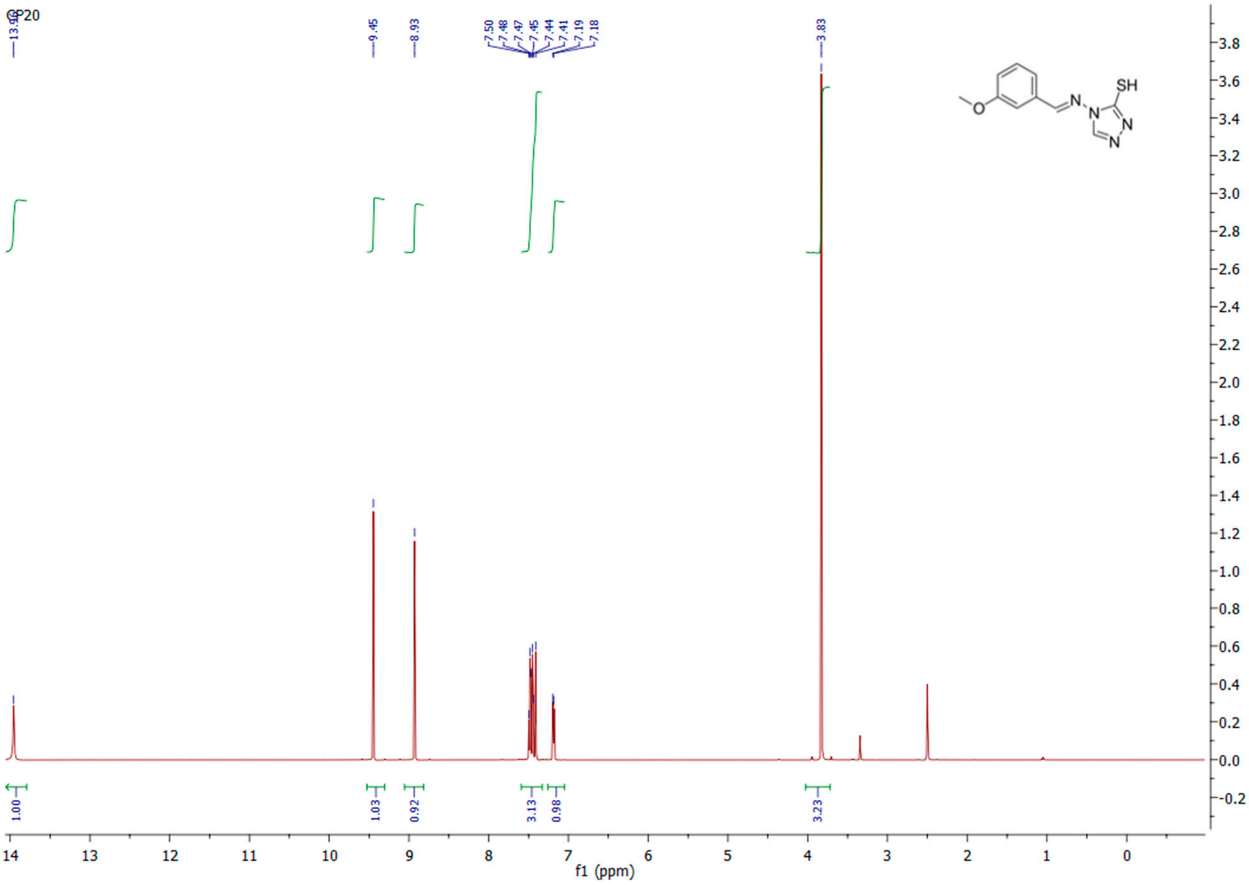
CP20
13.94
9.45
8.93
7.50
7.48
7.47
7.45
7.44
7.41
7.19
7.18
3.83
SH
N
N
N
N
O
1.00
1.03
0.92
3.13
0.98
3.23
f1 (ppm)

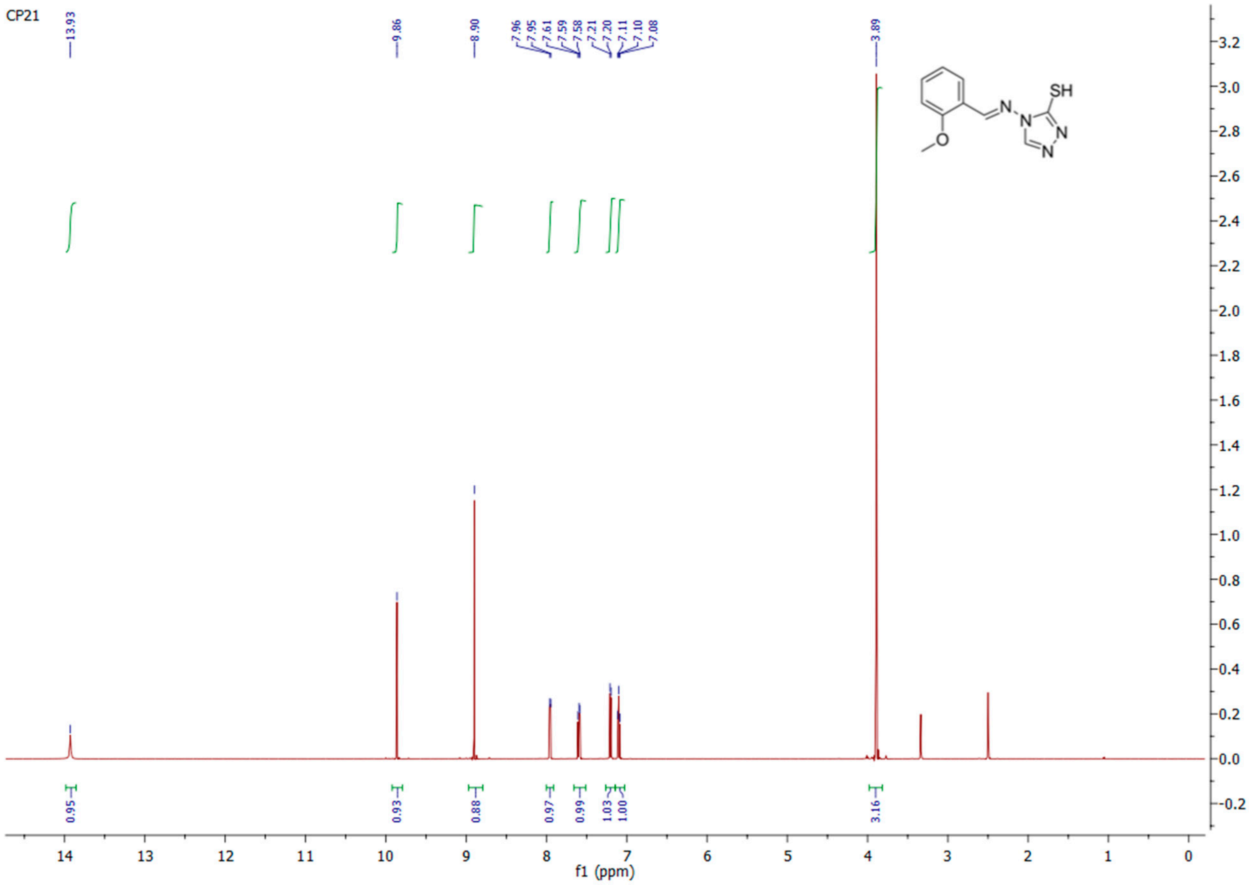
CP21
13.93
9.86
8.90
7.96
7.95
7.61
7.59
7.58
7.21
7.20
7.11
7.10
7.08
3.89
SH
N
N
N
N
O
0.95
0.93
0.88
0.97
0.99
1.03
1.00
3.16
f1 (ppm)

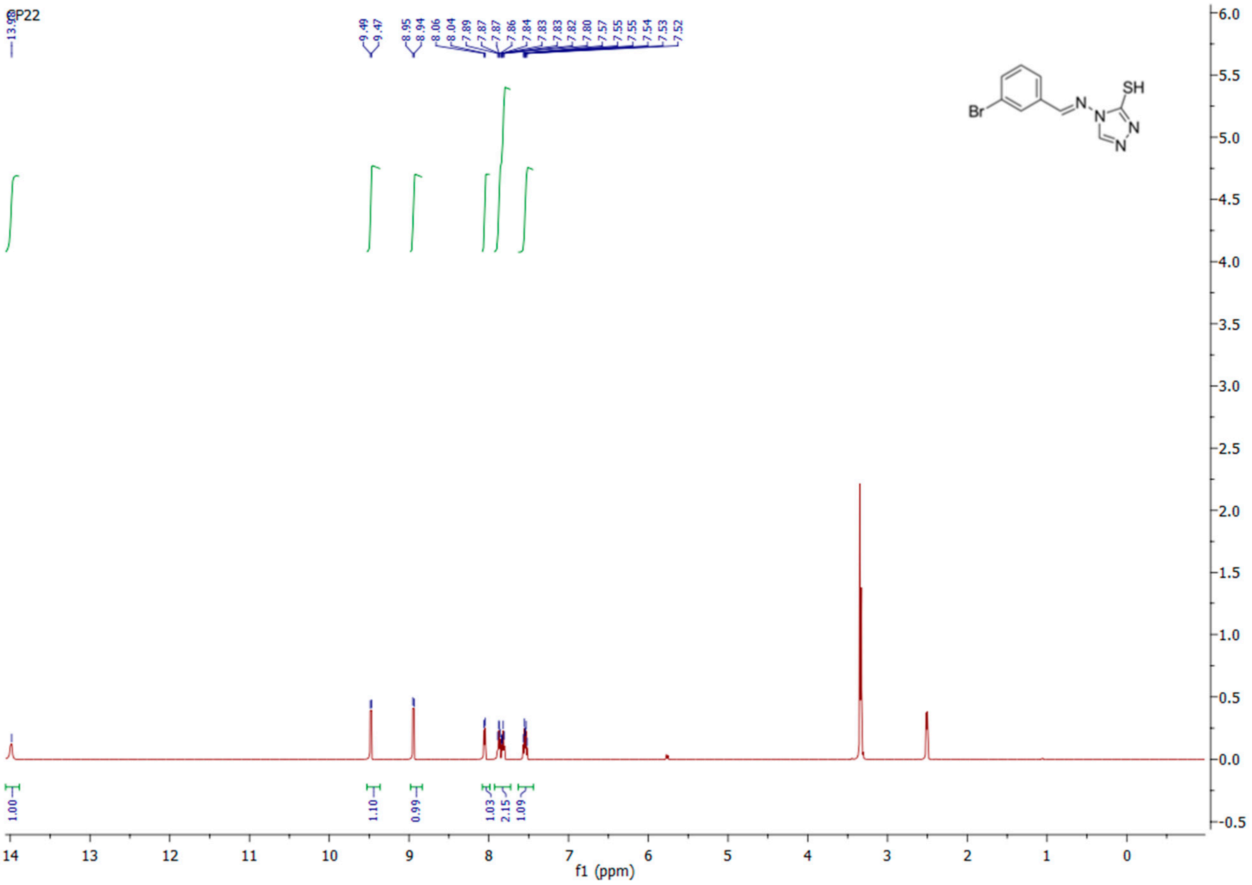
CP22
Br
N
SH
N
N
N
f1 (ppm)

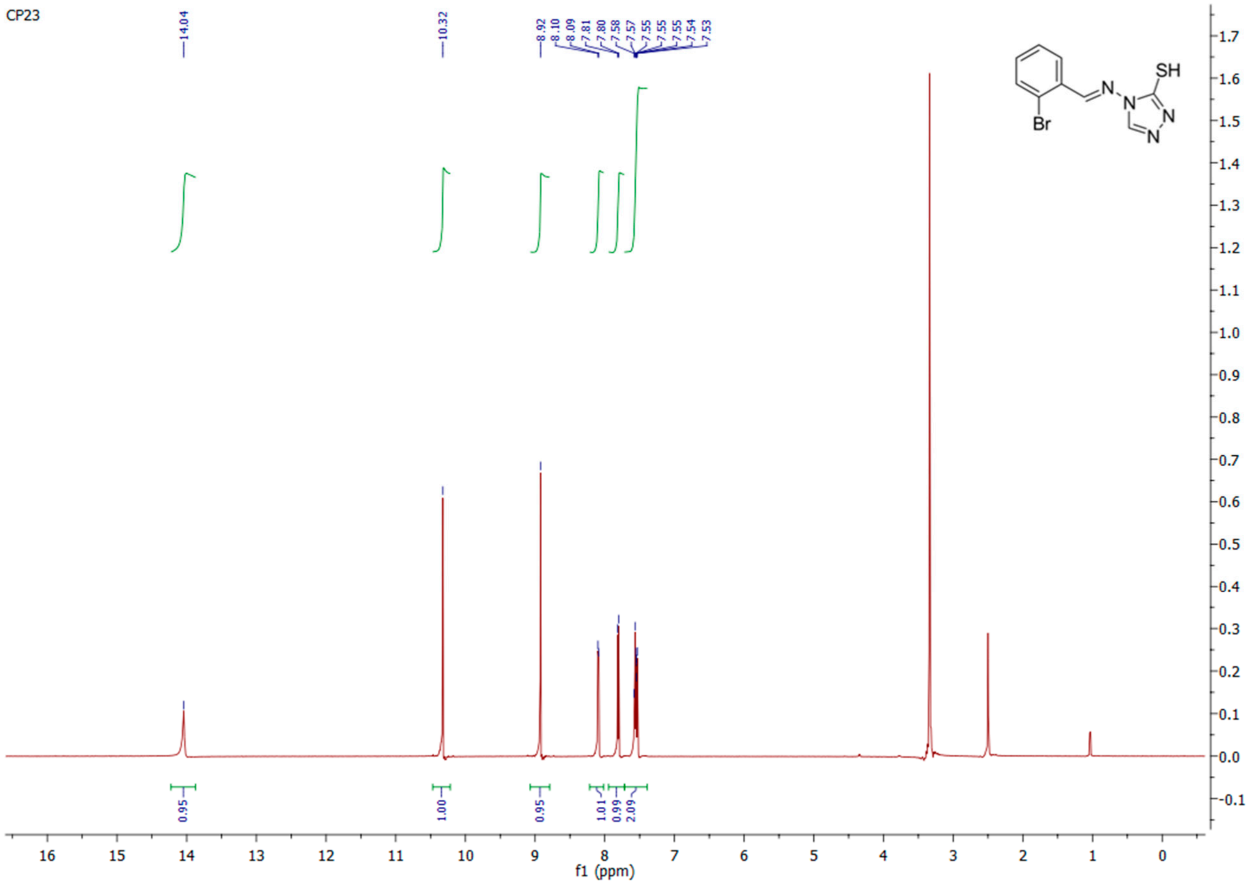
CP23
Br
N
SH
N
N
N
f1 (ppm)

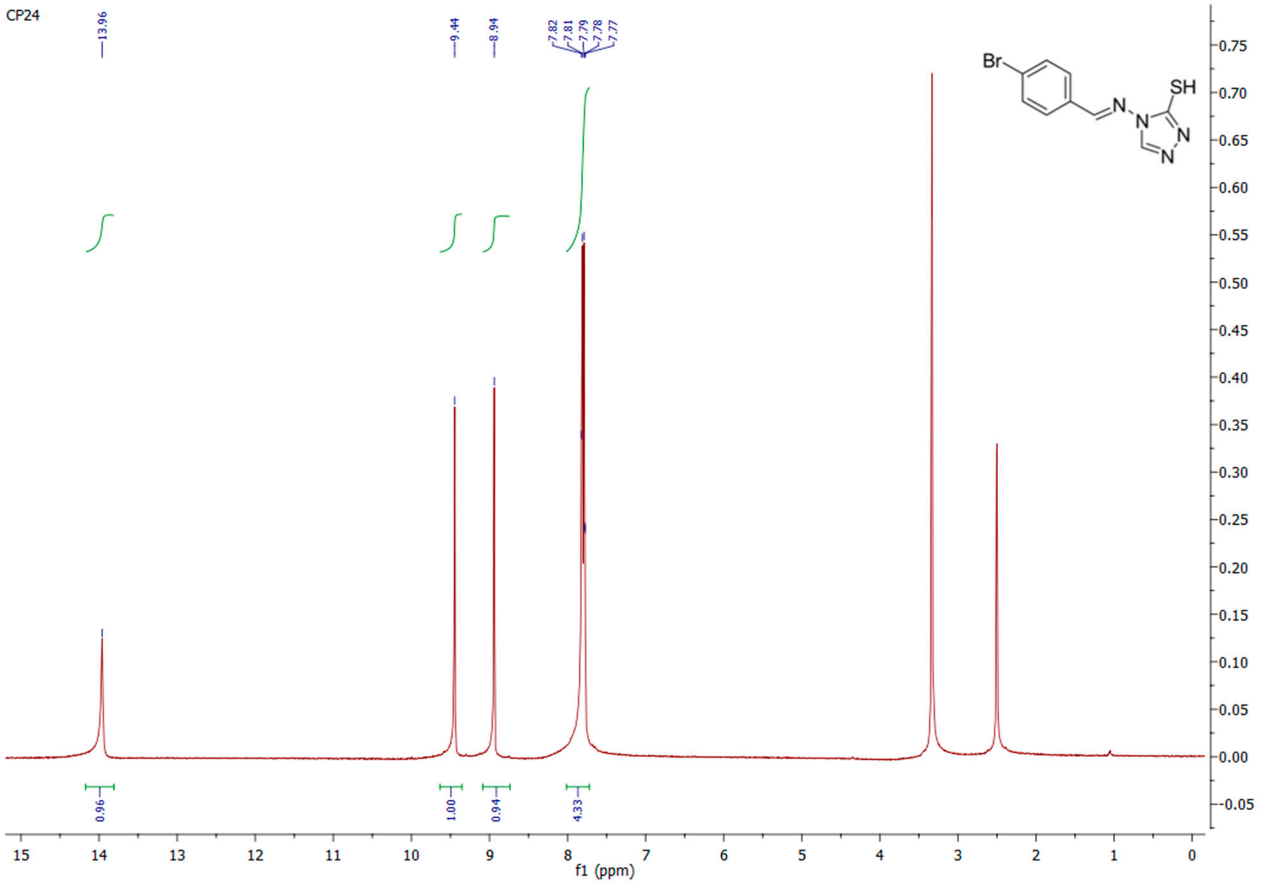
CP24
13.96
9.44
8.94
7.82
7.81
7.79
7.78
7.77
Br
N
SH
N
N
N
0.96
1.00
0.94
4.33
f1 (ppm)

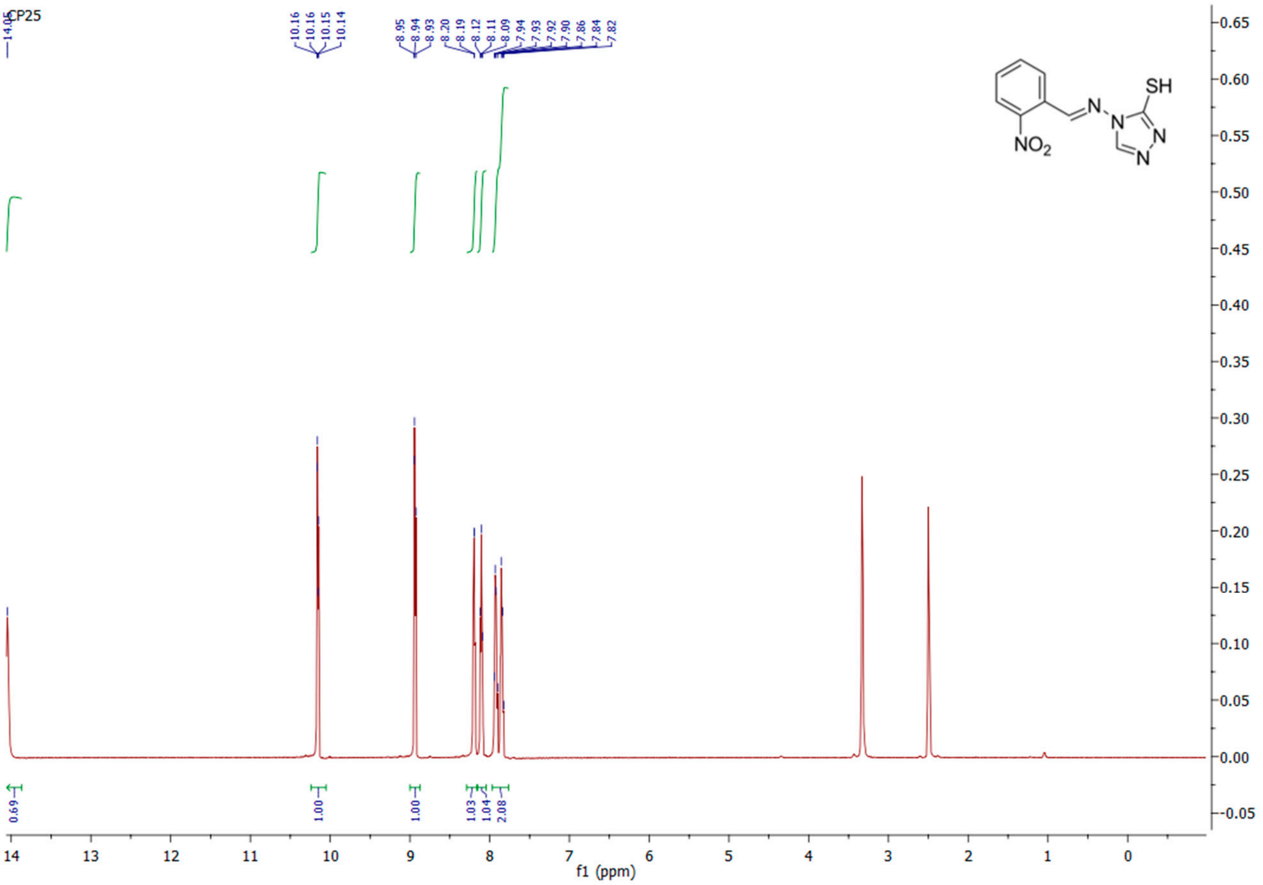
CP25
14.05
10.16
10.16
10.15
10.14
8.95
8.94
8.93
8.20
8.19
8.12
8.11
8.09
7.94
7.93
7.92
7.90
7.86
7.84
7.82
NO2
N
SH
N
N
N
0.69
1.00
1.00
1.03
1.04
2.08
f1 (ppm)

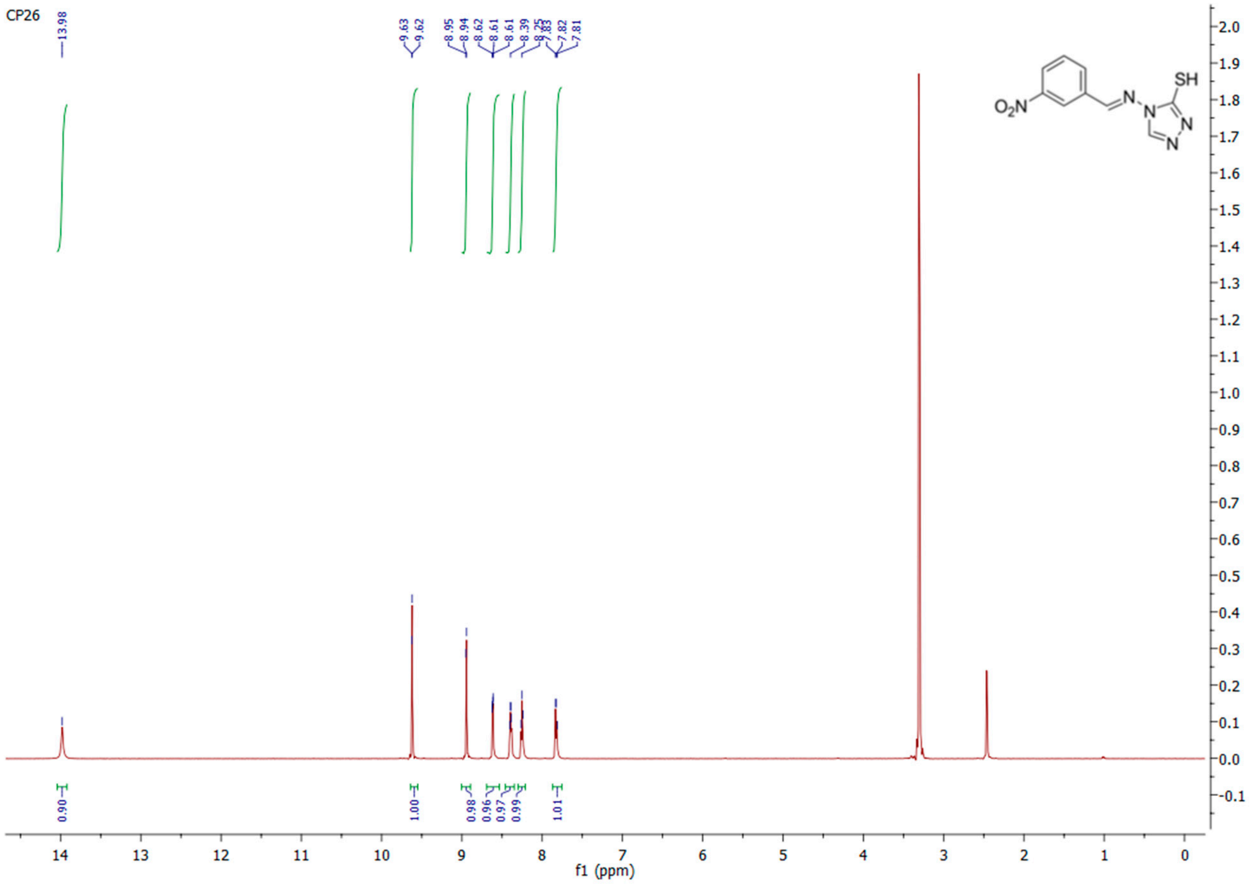
CP26
13.98
9.63
9.62
8.95
8.94
8.62
8.61
8.39
7.82
7.81
0.90
1.00
0.98
0.96
0.97
0.99
1.01
f1 (ppm)
SH
O_2N
N
N
N
N

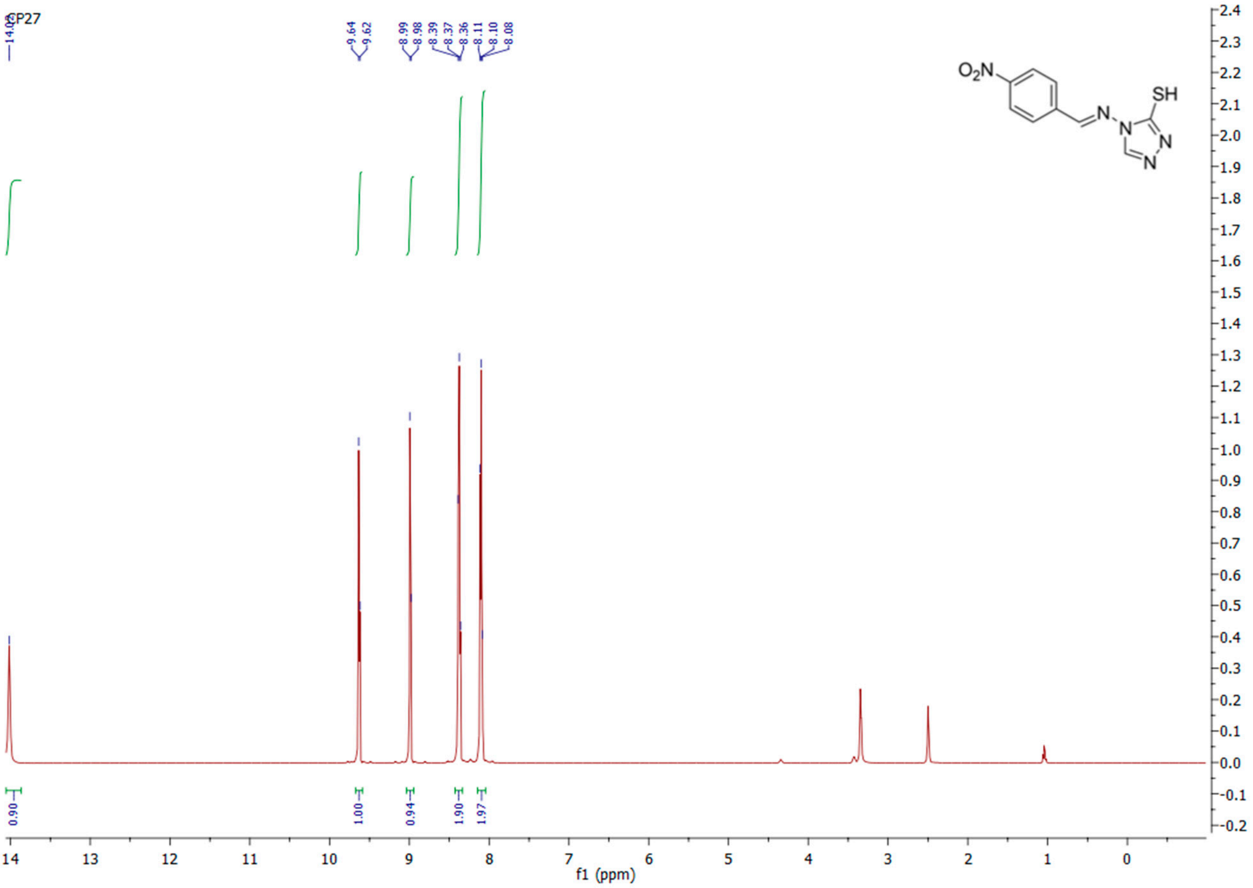
CP27
9.64
9.62
8.99
8.98
8.39
8.37
8.36
8.11
8.10
8.08
0.90
1.00
0.94
1.90
1.97
f1 (ppm)
SH
O_2N
N
N
N
N

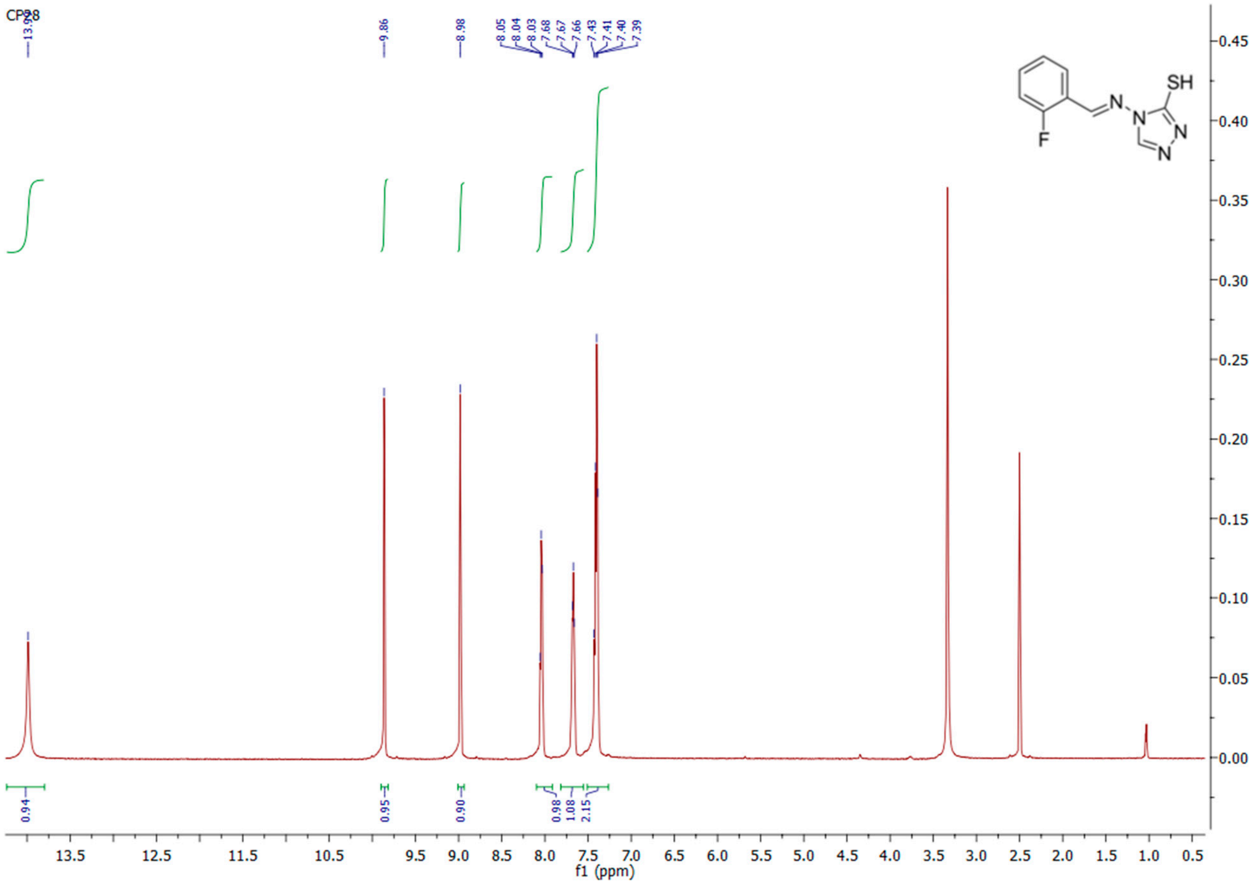
CP28
13.98
9.86
8.98
8.05
8.04
8.03
7.68
7.67
7.66
7.43
7.41
7.40
7.39
SH
N
F
0.94
0.95
0.90
0.98
1.08
2.15
f1 (ppm)

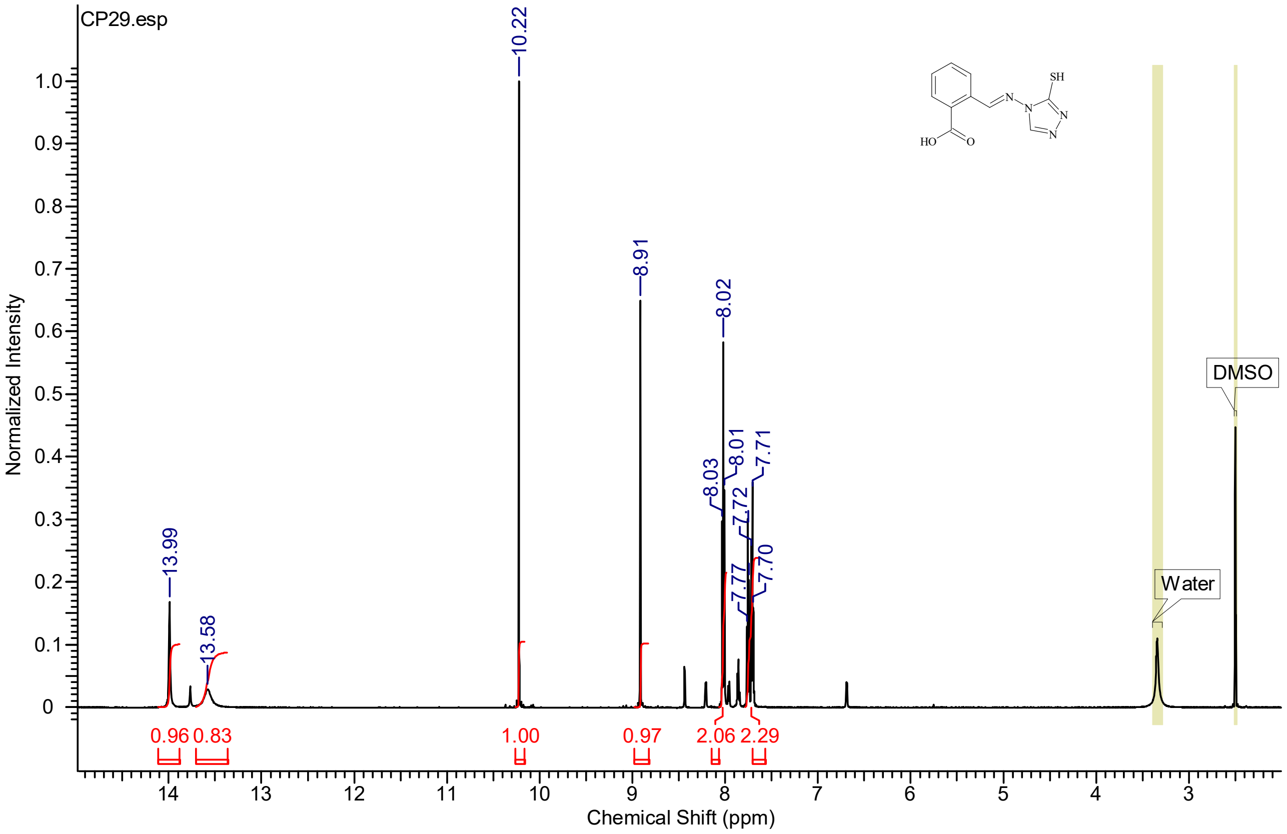
CP29.esp
10.22
8.91
8.02
8.03
8.01
7.72
7.71
7.77
7.70
13.99
13.58
SH
N
HO
O
DMSO
Water
Normalized Intensity
0.96
0.83
1.00
0.97
2.06
2.29
Chemical Shift (ppm)

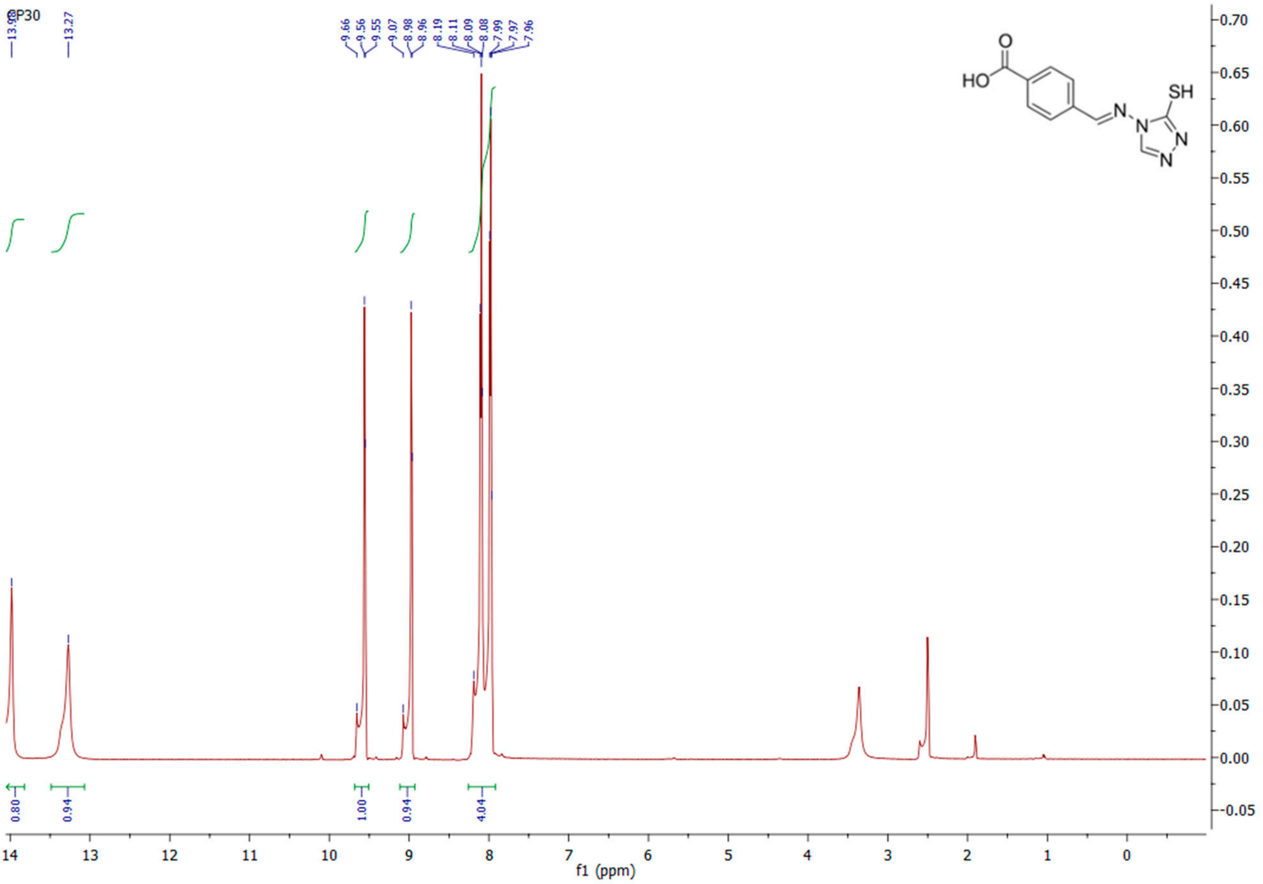
CP30
HO
O
N
N
SH
N
N
f1 (ppm)

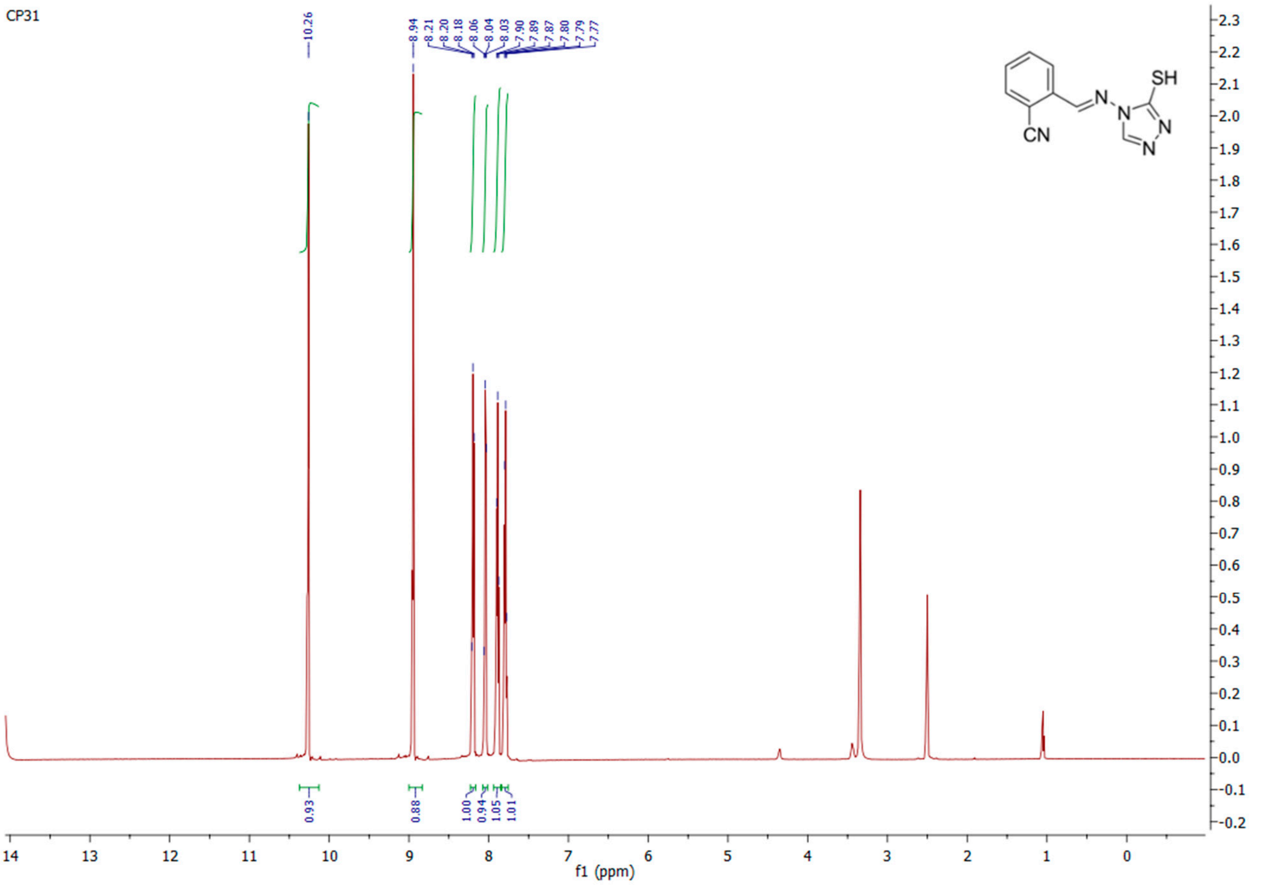
CP31
CN
N
N
SH
N
N
f1 (ppm)

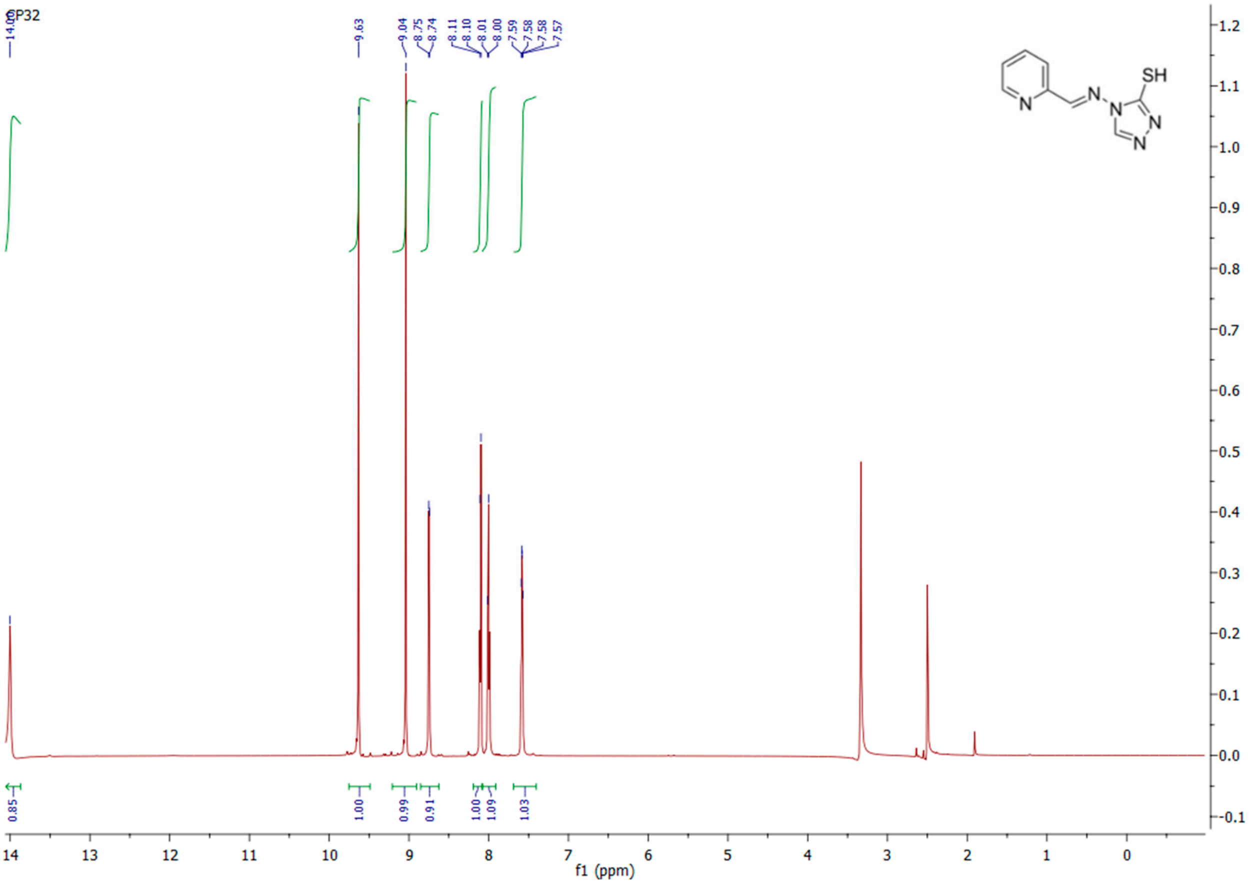

CP32
14.00
9.63
9.04
8.75
8.74
8.11
8.10
8.01
8.00
7.59
7.58
7.58
7.57
SH
N
0.85
1.00
0.99
0.91
1.00
1.09
1.03
f1 (ppm)

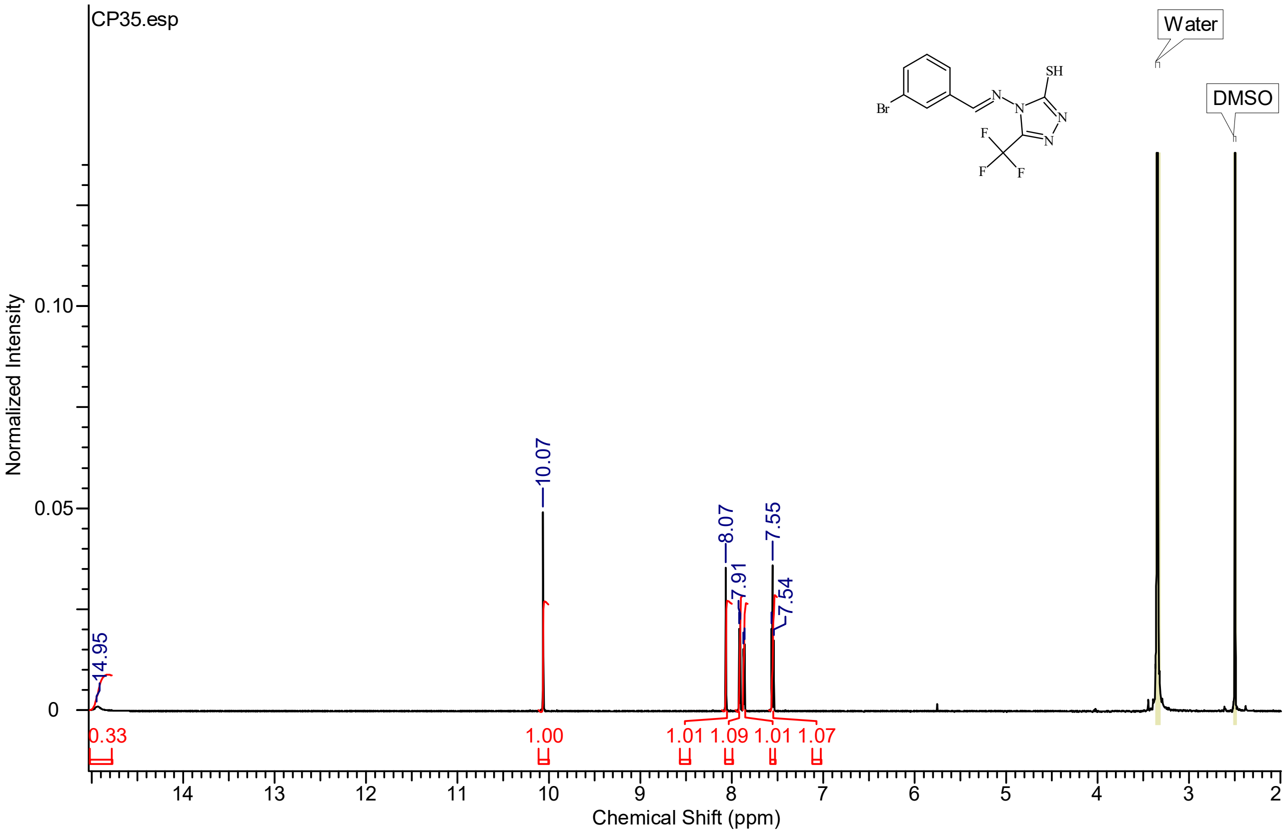

CP35.esp
Water
DMSO
SH
Br
N
F
Normalized Intensity
14.95
10.07
8.07
7.91
7.55
7.54
0.33
1.00
1.01
1.09
1.01
1.07
Chemical Shift (ppm)

CP44

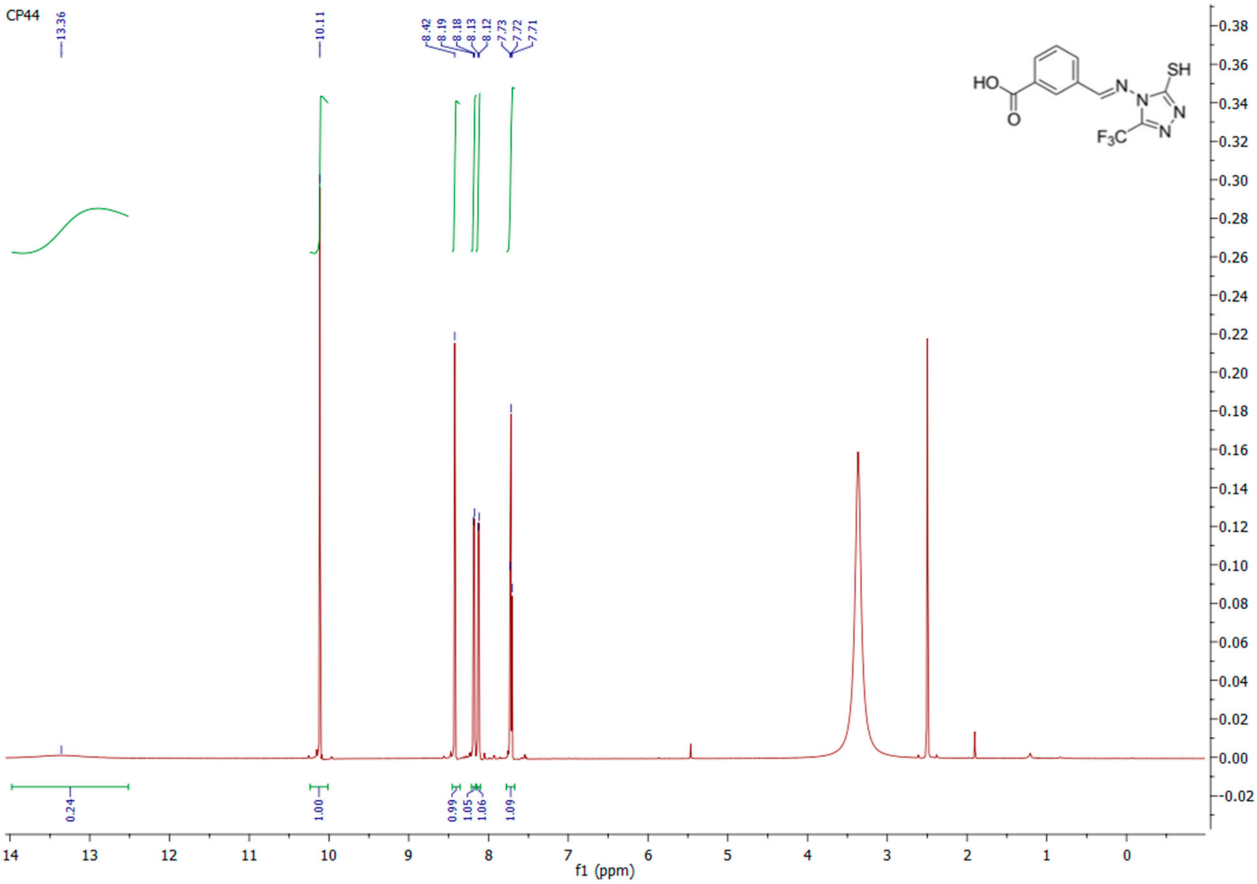

CP45

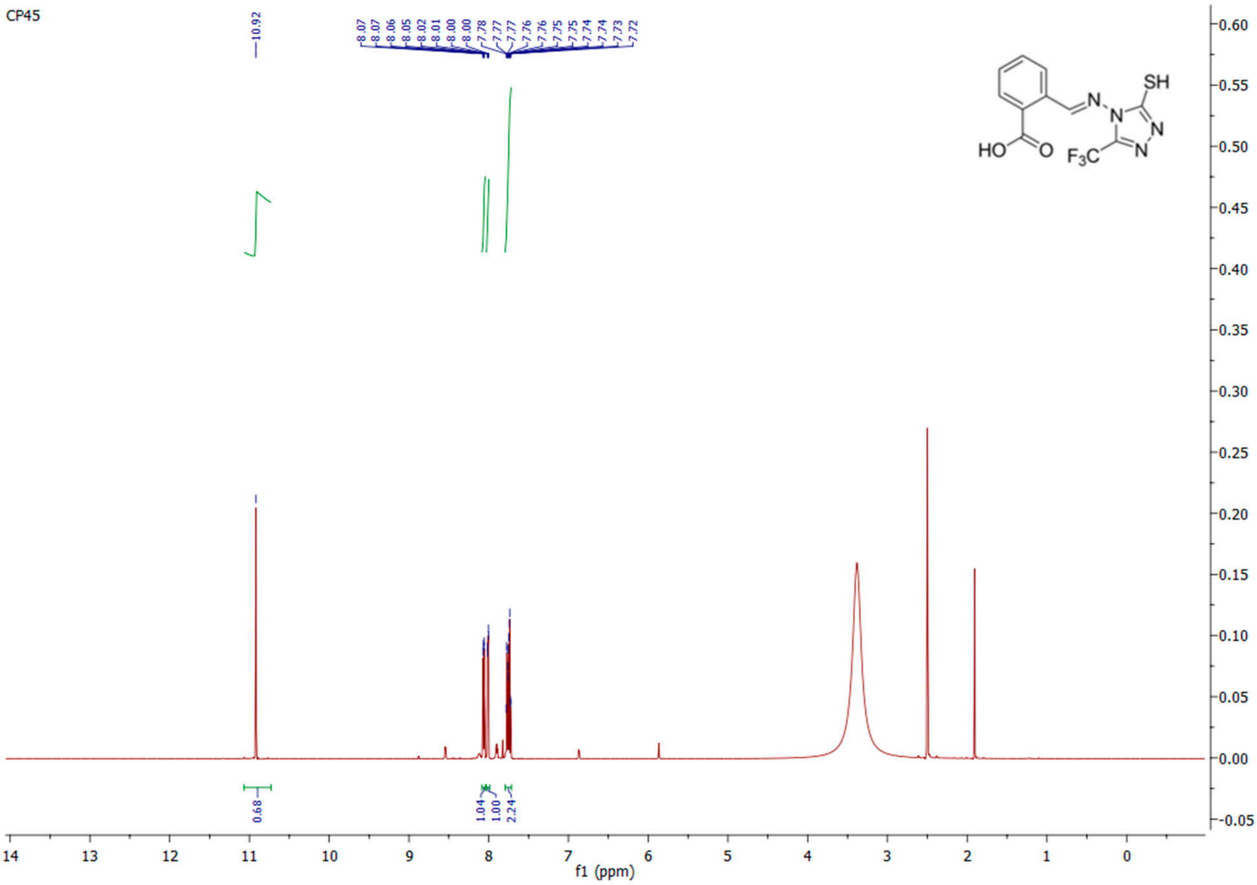

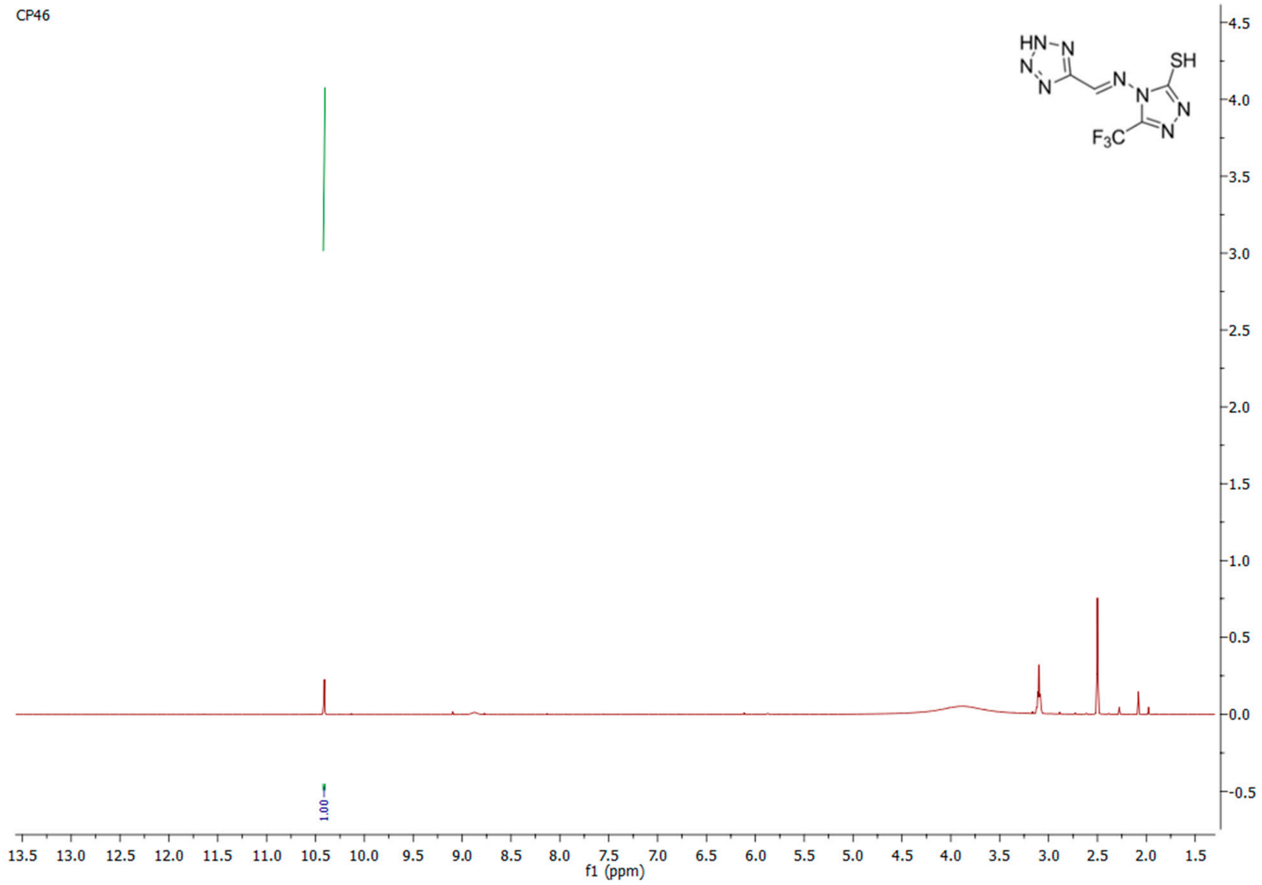
CP46
f1 (ppm)
1.00
SH
F3C
HN
N

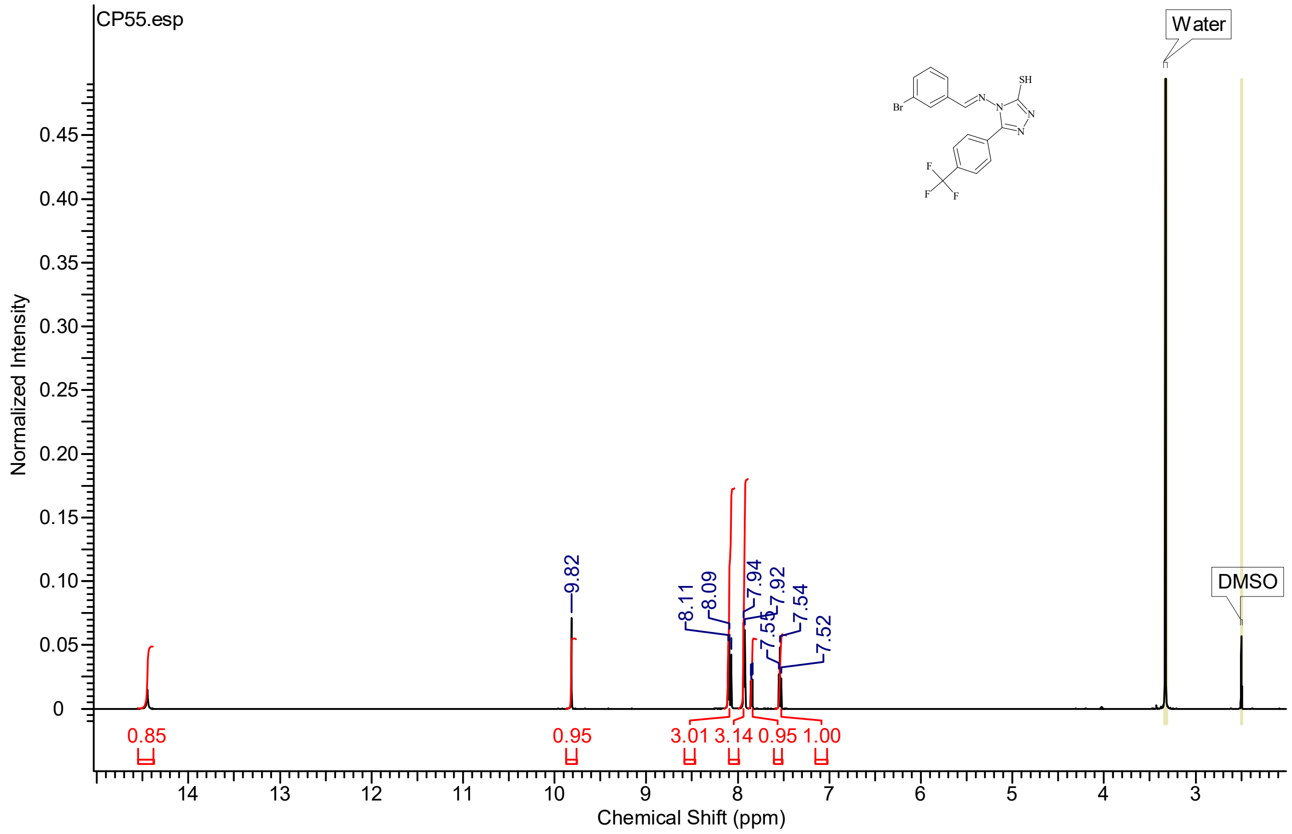
CP55.esp
Water
DMSO
Normalized Intensity
Chemical Shift (ppm)
9.82
8.11
8.09
7.94
7.92
7.55
7.54
7.52
0.85
0.95
3.01
3.14
0.95
1.00
SH
Br

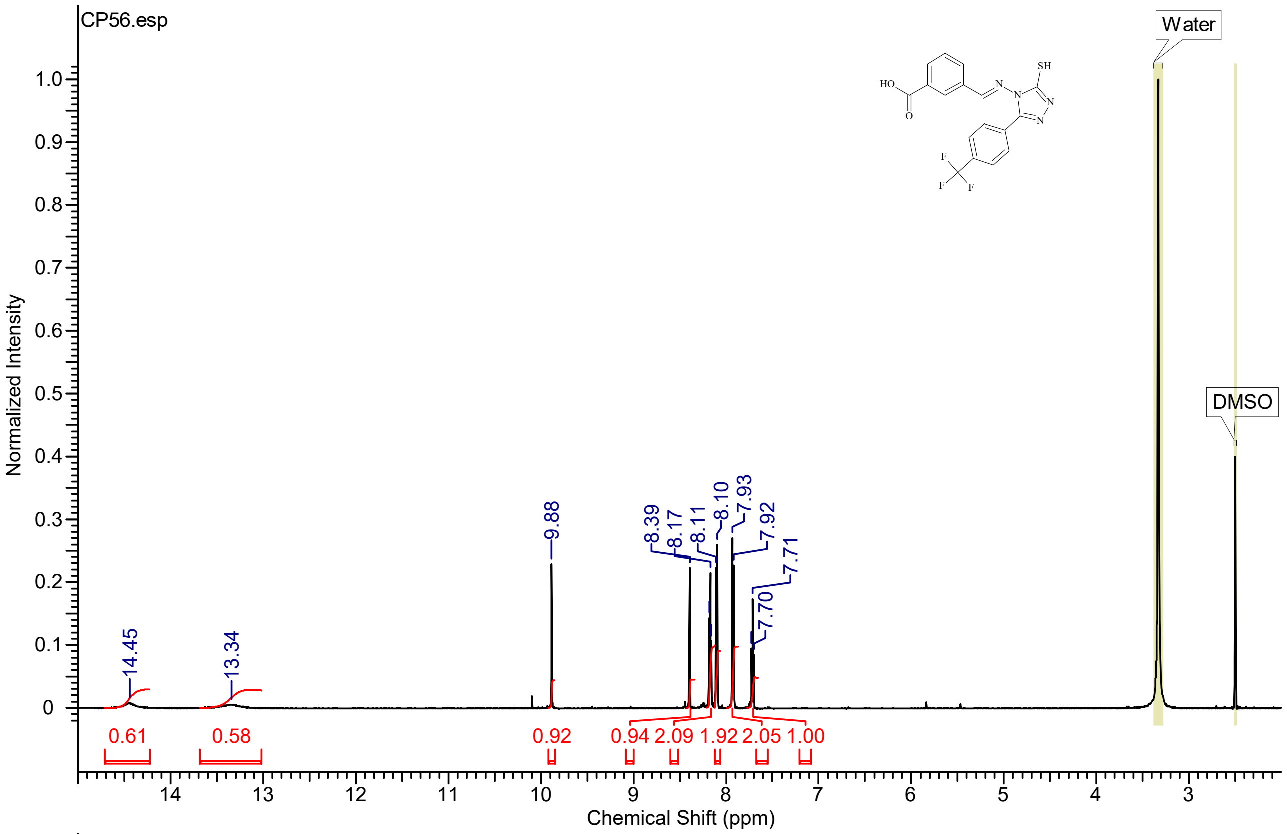
CP56.esp
Water
DMSO
Normalized Intensity
Chemical Shift (ppm)
14.45
13.34
9.88
8.39
8.17
8.11
8.10
7.93
7.92
7.71
7.70
0.61
0.58
0.92
0.94
2.09
1.92
2.05
1.00

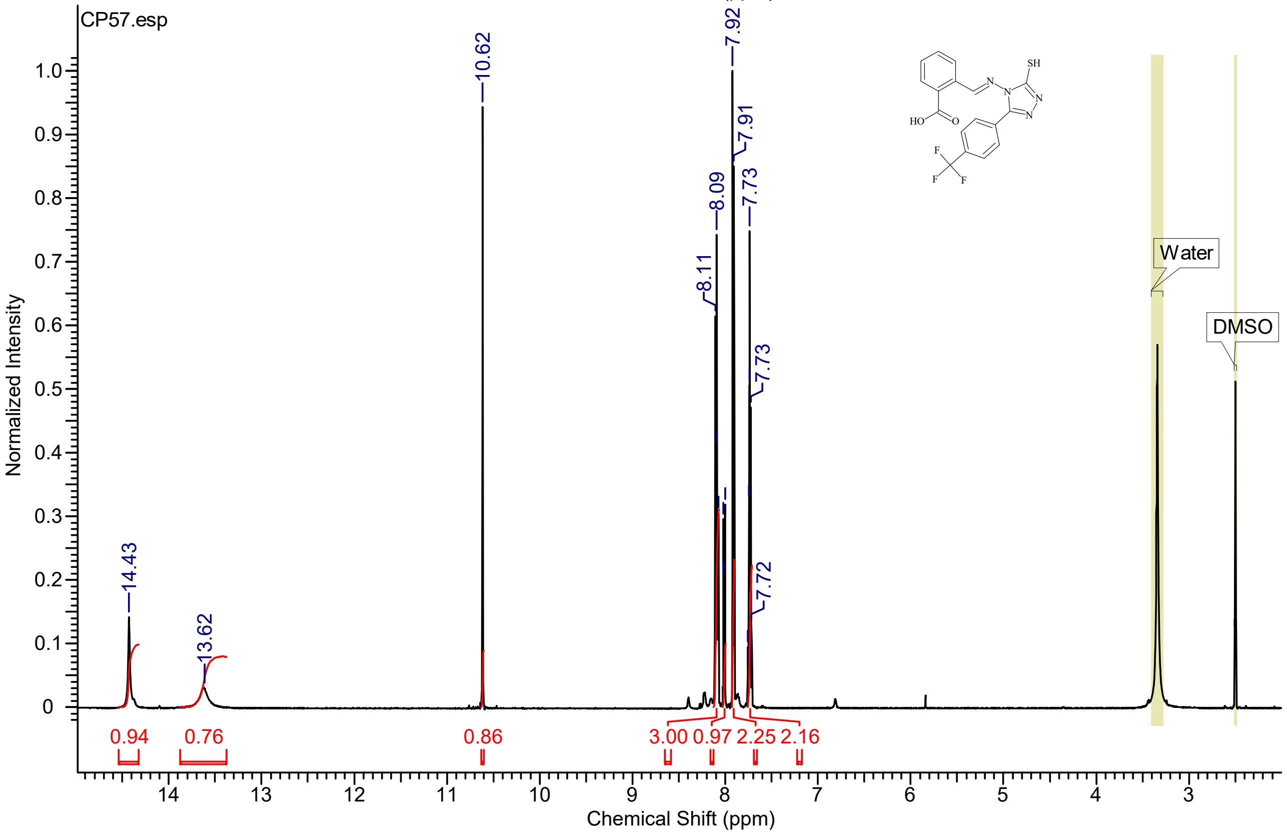
CP57.esp
Water
DMSO
Normalized Intensity
Chemical Shift (ppm)
14.43
13.62
10.62
8.11
8.09
7.92
7.91
7.73
7.73
7.72
0.94
0.76
0.86
3.00
0.97
2.25
2.16

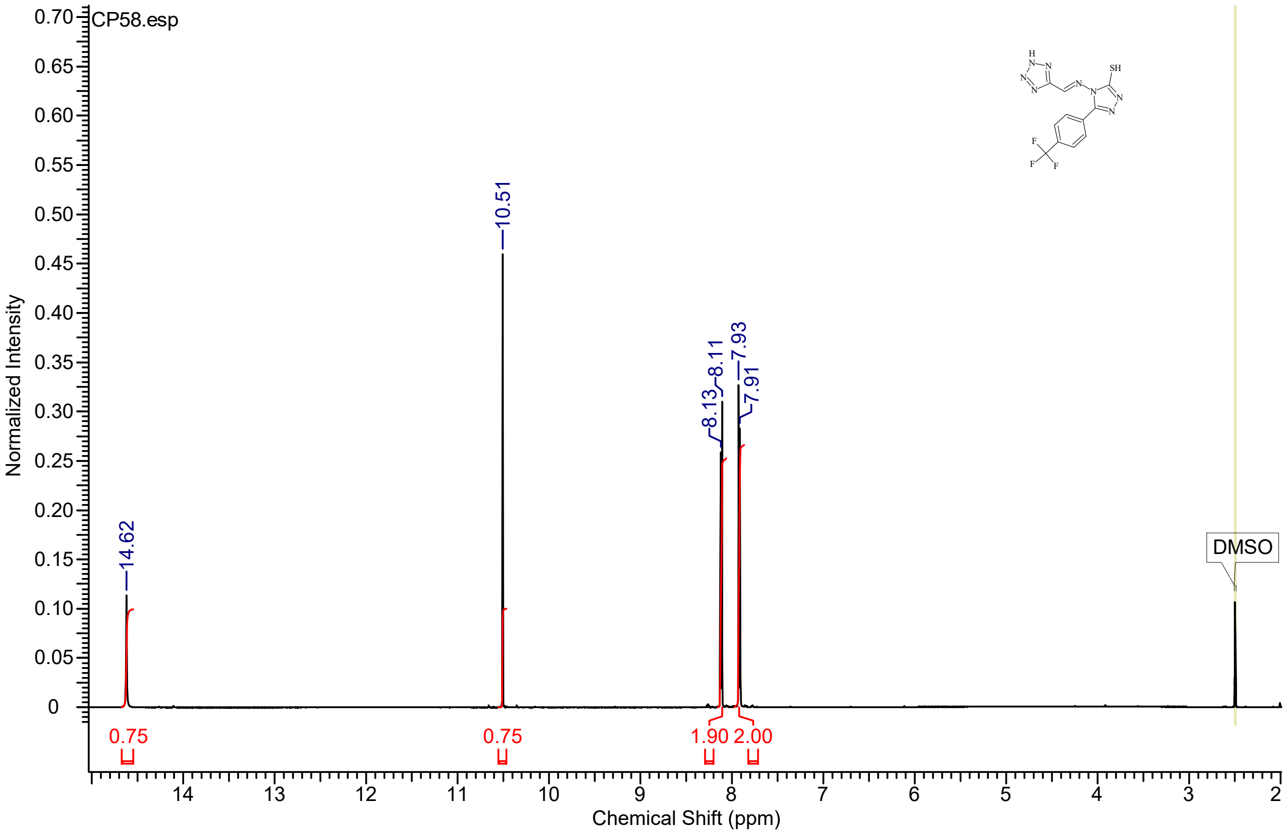
CP58.esp
Normalized Intensity
Chemical Shift (ppm)
14.62
10.51
8.13
8.11
7.93
7.91
DMSO
0.75
0.75
1.90
2.00
SH
F
F
F

Figure S3. Structure-Activity relationship for synthesized compounds

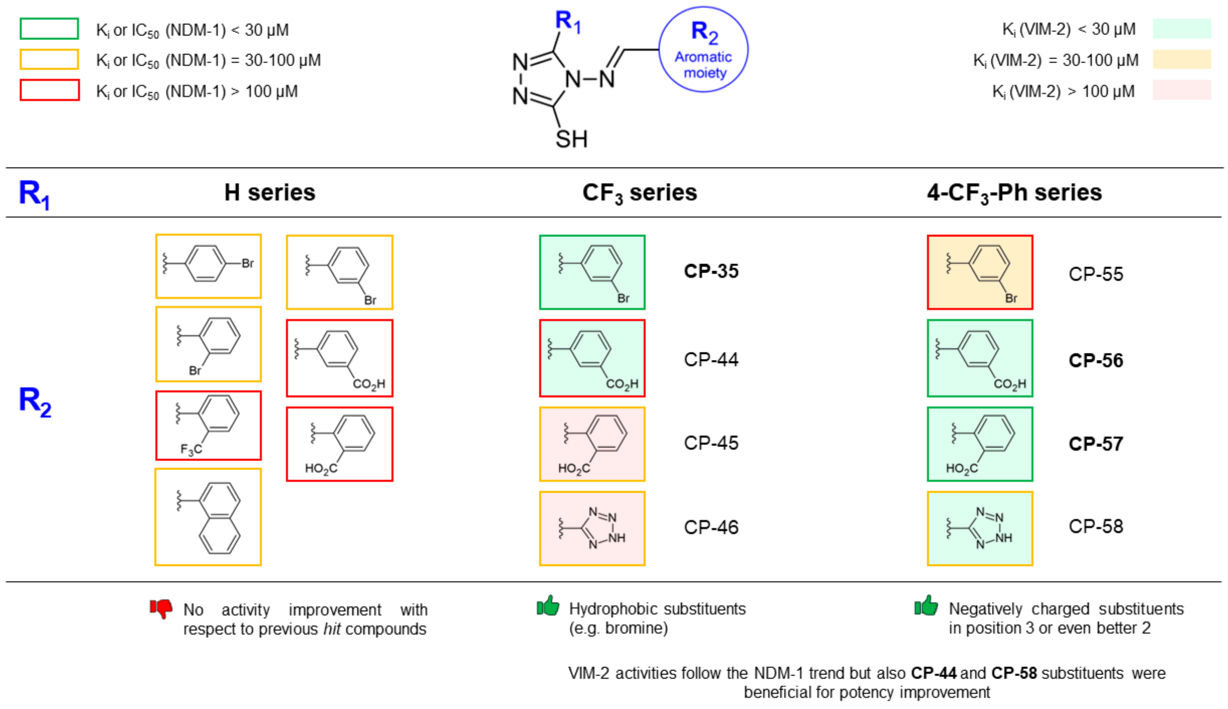

Figure S4. Docking procedure validation result for compound OP31

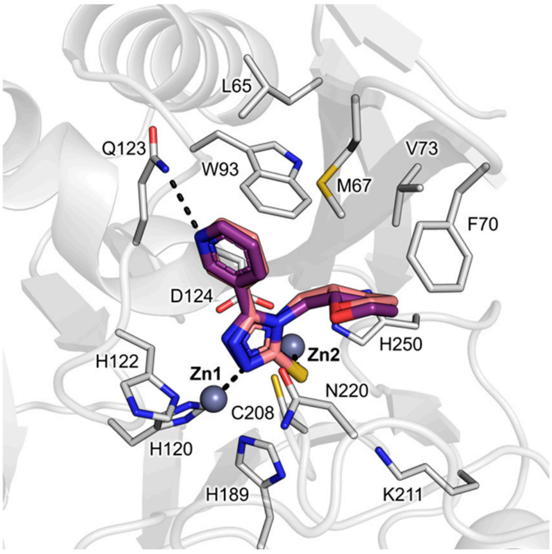

Supplement: Supplementary file 1 [file pharmaceuticals-16-01682-s001.zip › pharmaceuticals-2728467-supplementary.pdf]
